# Supplementary material for: Design of target specific peptide inhibitors using generative deep learning and molecular dynamics simulations
Source: Nat Commun. 2024 Feb 21;15:1611. doi: 10.1038/s41467-024-45766-2 (PMC10882002; doi:10.1038/s41467-024-45766-2)
Supplement: Supplementary file 1 — Supplementary Information [file 41467_2024_45766_MOESM1_ESM.pdf]

SUPPLEMENTARY INFORMATION
for
Design of peptide inhibitors using generative deep learning and molecular dynamics simulations

Sijie Chen[1†], Tong Lin[2,3†], Ruchira Basu[4], Jeremy Ritchey[4], Shen Wang[1], Yichuan Luo[5], Xingcan Li[6], Dehua Pei[4*], Levent Burak Kara[2*] and Xiaolin Cheng[1,7*]

[1]College of Pharmacy, The Ohio State University, 281 W Lane Ave, Columbus, 43210, OH, United States.

[2]Mechanical Engineering Department, Carnegie Mellon University, 5000 Forbes Ave, Pittsburgh, 15213, PA, United States.

[3]Machine Learning Department, Carnegie Mellon University, 5000 Forbes Ave, Pittsburgh, 15213, PA, United States.

[4]Department of Chemistry and Biochemistry, The Ohio State University, 281 W Lane Ave, Columbus, 43210, OH, United States.

[5]Electrical and Computer Engineering Department, Carnegie Mellon University, 5000 Forbes Ave, Pittsburgh, 15213, PA, United States.

[6]Department of Radiology, Affiliated Hospital and Medical School of Nantong University, 20 West Temple Road, Nantong, 226001, Jiangsu, China.

[7]Translational Data Analytics Institute, The Ohio State University, 1760 Neil Ave, Columbus, 43210, OH, United States.

*Corresponding author(s). E-mail(s): pei.3@osu.edu; lkara@cmu.edu; cheng.1302@osu.edu

†These authors contributed equally to this work.

Supplementary Tables

Supplementary Table 1: MM/GBSA and IC_{50} results of 14 experimentally tested peptides.*

Peptide	Peptide sequence	MM/GBSA (kcal/mol)	***In vitro*** **IC_{50} (μM)**
1	GGYPEDILDKHLQRZIL	-33.0 ± 1.2	0.1 ± 0.01
2	GGYPEDILDKHLQRZWL	-48.9 ± 4.4	0.071 ± 0.006
3	YPEDILDKHLQRZ(3-Bta)R	-46.8 ± 5.4	0.037 ± 0.02
4	GGYPEDILDKHLQRZ(2-Nal)L	-38.7 ± 3.9	0.087 ± 0.03
5	GGYPEDILDKHLQRZ(3-Bta)L	-43.6 ± 2.0	0.065 ± 0.02
6	GGYPEDILDKHLQRZ(3-Bta)R	-45.9 ± 1.2	0.037 ± 0.02
7	YPEDILDKHLQRZ(3-Bta)dR	-50.5 ± 5.0	0.041 ± 0.008
8	YPEDILDKHLQRV(dA)L	-46.1 ± 1.5	0.062 ± 0.006
9	GGYPEDILDKHLQRVIL	-35.4 ± 4.2	0.15 ± 0.04
10	GGYPEDILDKHLQRVI	-39.2 ± 2.1	1.5 ± 0.1
11	GGYPEDILDKHLQRV	-31.4 ± 3.8	1.5 ± 0.2
12	GGWPEDILDKHLQRV	-33.3 ± 2.6	5.2
13	GGWPEDILDKHVQRV	-33.2 ± 1.6	240
14	YPEDILDKHLQRVAL	-41.2 ± 3.9	0.11 ± 0.006

*Chemical structures of the unnatural amino acids 3-Bta, TertLeucine (Z) and 2-Nal are provided below:

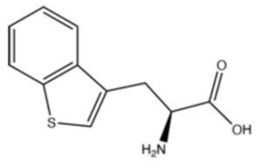

3-Bta

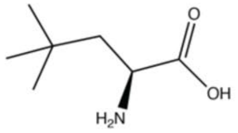

TertLeucine (Z)

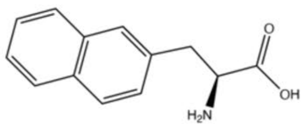

2-Nal

Supplementary Table 2: Performance comparison of the different machine learning models for estimating q(c|z).

a. N-terminus extension

Model	Accuracy (with/without bootstrap)
**Support vector classifier**	85.6% / 82.3%

**Logistic regression**	61.4% / 61.6%
**Extreme gradient boosting tree**	71.6% / 69.8%

b. C-terminus extension

Model	Metric	Pretrain (with/without bootstrap)	finetune 1 (with/without bootstrap)	finetune 2 (with/without bootstrap)	finetune 3 (with/without bootstrap)
**Support vector classifier**	Accuracy	84.3% / 81.1%	81.3% / 80.2%	85.2% / 84.5%	88.0%/88.6%
	Precision	93.3%/87.7%	81.2%/80.0%	74.5%/ 73.2%	76.2%/75.3%
**Logistic regression**	Accuracy	53.3% / 51.3%	78.4/79.3%	70.9%/69.1%	86.9%/85.1%
	Precision	50% / 0%	45.5%/50%	14.5%/9.66%	12.5%/8.33%
**Extreme gradient boosting tree**	Accuracy	82.6% / 80.8%	89.3% / 90.0%	84.6%/84.8%	91.0%/89.6%
	Precision	100% / 100%	100% /100%	92.6%/79.6%	100%/81.5%
**K nearest neighbor**	Accuracy	66.5% / 64.3%	88.4%/86.0%	70.0%/64.2%	72.1%/88.2%
	Precision	47.4%/100%	80.0%/16.7%	10.5%/31.2%	12.5%/28.6%

Supplementary Table 3: MM/GBSA results of N-terminally extended peptides optimized with SA (kcal/mol).

**Peptide sequence**	**MM/GBSA run1**	**MM/GBSA run2**	**MM/GBSA run3**	**MM/GBSA mean**
SSFRPYPEDILDKHLQRVIL	-45.0	-43.3	-37.3	-41.8
SSFSPYPEDILDKHLQRVIL	-37.5	-37.3	-30.5	-35.1
SWFRPYPEDILDKHLQRVIL	-30.4	-40.7	-37.4	-36.1
SSFSYYPEDILDKHLQRVIL	-29.0	-47.2	-30.2	-35.4

Supplementary Table 4: MM/GBSA results of N-terminally extended peptides optimized with GA (kcal/mol).

**Peptide sequence**	**MM/GBSA run1**	**MM/GBSA run2**	**MM/GBSA run3**	**MM/GBSA mean**
WLPYPEDILDKHLQRVIL	-29.3	-38.0	-28.7	-32.0
WFPYPEDILDKHLQRVIL	-33.7	-42.1	-40.0	-38.6

RKPYPEDILDKHLQRVIL	-29.8	-34.0	-39.5	-34.4
LTPYPEDILDKHLQRVIL	-31.7	-40.5	-39.2	-37.1
LKPYPEDILDKHLQRVIL	-32.3	-34.8	-37.2	-34.8
TIPYPEDILDKHLQRVIL	-33.0	-45.8	-30.5	-36.4
LHPYPEDILDKHLQRVIL	-35.8	-44.8	-29.3	-36.6
HRNDYPEDILDKHLQRVIL	-52.6	-50.6	-43.1	-48.7
RLRDYPEDILDKHLQRVIL	-49.0	-36.7	-45.7	-43.8
HRRDYPEDILDKHLQRVIL	-41.8	-46.0	-36.8	-41.5
SIRDYPEDILDKHLQRVIL	-36.0	-40.5	-44.5	-40.3

Supplementary Table 5: Sequences of hit peptides from OBOC library screening.

Hit No.	X1	X2	X3	X4
L1	D-Trp	cis-AcPc	D-Thr	Orn
L2*	D-Trp	D-β-hA	Orn	Orn
L3	Ser	D-Asn	D-Nal	D-Arg
L4	D-Arg	Ala	isoAsp	D-Asn
L5	cis-Acpc	D-Trp	His	Orn
L6	Gln	D-Nal	D-Ala	D-Glu
L7	His	Orn	Phg	Nle
L8	D-Arg	D-Ala	D-β-hA	D-Arg
L9	Gln	Orn	D-Thr	Gln
L10	D-Arg	D-Arg	Gly	D-Leu
L11	D-Tyr	Fpa	Asp	D-Arg
L12*	D-Thr	Gln	D-Arg	Gln
L13	cis-Acpc	D-Thr	D-Tyr	D-Arg
L14	D-Tyr	D-Arg	Fpa	Gly
L15	cis-Acpc	Ser	D-Tyr	Orn
L16	D-Trp	Fpa	D-Thr	D-Arg
L17*	D-Lys	D-Ala	Ile	D-Arg
L18	Phg	Ile	D-Thr	D-Lys
L19*	D-Val	D-Asn	D-Lys	D-Arg

L20*	D-Arg	Nle	Ser	Orn
L21	Gly	β-Ala	D-Arg	D-Arg
L22	D-Tyr	Gln	Orn	D-Asn
L23	Nle	Ile	D-Asn	Fpa
L24	Ile	Phg	D-Arg	Nle

Supplementary Table 6: Peptides selected from library screening and their IC_{50} results for β-catenin. All peptides contain the same C-terminal sequence YPEDILDKHLQRV-NH_2.

**Peptide ID**	**N-terminal Sequence (OBOC Library)**				***In vitro* IC_{50} (μM)**
	**X1**	**X2**	**X3**	**X4**	
Parent	Null	Null	Gly	Gly	-48.9 ± 4.4
L2	D-Trp	D-hAla	Orn	Orn	2.2 ± 0.5
L12	D-Thr	Gln	Orn	Orn	2.8 ± 0.1
L17	D-Lys	D-Ala	Ile	D-Arg	3.9 ± 0.1
L19	D-Val	D-Asn	D-Lys	D-Arg	3.7 ± 0.5
L20	D-Arg	Nle	Ser	Orn	1.5 ± 0.1

Supplementary Methods

Supplementary Method 1: Algorithm for updating proposal distribution.

Algorithm 1 UpdateQ

Require: Update the parameters of the proposal distribution

Input: classifier q(c= potential binder | z), encoding distribution q(z), mean of proposal distribution μ, newly proposed extension encoding $\tilde{z}_1, z, \tilde{z}$

Output: Decoded peptide extension

1. Compute $A = \min(0, \log q(c|\tilde{z}) + \log q_z(\tilde{z}) - \log q(c|z) - \log q(z))$
2. Sample u from U(0,1)
3. **if** $\log u \le A$ ***then***
4. Set $\mu = \tilde{z}_1$
5. **end if**
6. **Return** μ

Supplementary Method 2: Algorithm for peptide extension sampling.

Algorithm 2 Conditional sequence sampling

Require: Sampling z_1 from q(z_1|c, z_2)

Input: classifier q(c|z), encoding distribution q(z), extension decoder D1, base encoder E2, base sequence x_{base}, label c = potential binder,

Output: Decoded peptide extension

1. **Initialization**, $z_1^0 = \mu_1^0 = mean\ of\ z_1\ of\ train\ potential\ binder, \sigma = 0.05 \times std\ of\ z_1\ of\ train\ potential\ binder, Q(z_1^1 | z_1^0) = Gauss(\mu_1^0, \sigma), N = 0, N_{seq} = 0, N_{desired} = 100, N_{burn-in} = 500, N_{limits} = 200, empty\ list\ SeqList$
2. $z_2 = E2(x_{base})$
3. $z^0 = [z_1^0, z_2]$
4. **while** $N < N_{burn-in}$ **do**
5. $Sample\ \tilde{z}_1\ from\ Gauss(\mu_1^N, \sigma)$
6. $\tilde{z} = [\tilde{z}_1, z_2]$
7. $\mu_1^{N+1} = UpdateQ\ (q_c, q_z, \mu_1^N, \tilde{z}_1, z^N, \tilde{z})$
8. $z_1^{N+1} = \mu_1^{N+1}$
9. $z^{N+1} = [z_1^{N+1}, z_2]$
10. $N = N + 1$
11. **end while**
12. **while** $N_{seq} < N_{desired}$ and $N \leq N_{limits} + N_{burn-in}$ **do**
13. $Sample\ \tilde{z}_1\ from\ Gauss(\mu_1^N, \sigma)$
14. $\tilde{z} = [\tilde{z}_1, z_2]$
15. $\mu_1^{N+1} = UpdateQ\ (q_c, q_z, \mu_1^N, \tilde{z}_1, z^N, \tilde{z})$
16. $z_1^{N+1} = \mu_1^{N+1}$
17. $z^{N+1} = [z_1^{N+1}, z_2]$
18. $Extension = D1(\ z_1^{N+1})$
19. **if** Extension is not in SeqList **then**
20. Add Extension into SeqList
21. $N_{seq} = N_{seq} + 1$
22. **end if**
23. $N = N + 1$
24. **end while**

Supplementary Figures

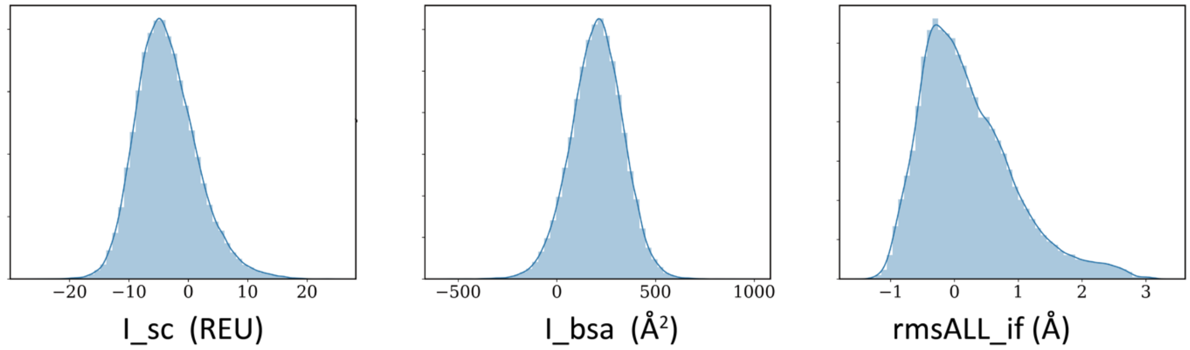

Supplementary Figure 1: Probability distributions of the three Rosetta FlexPepDock scores I_sc, I_bsa and rmsALL_if for peptides generated from Rosetta Design. Source data are provided as a Source Data file.

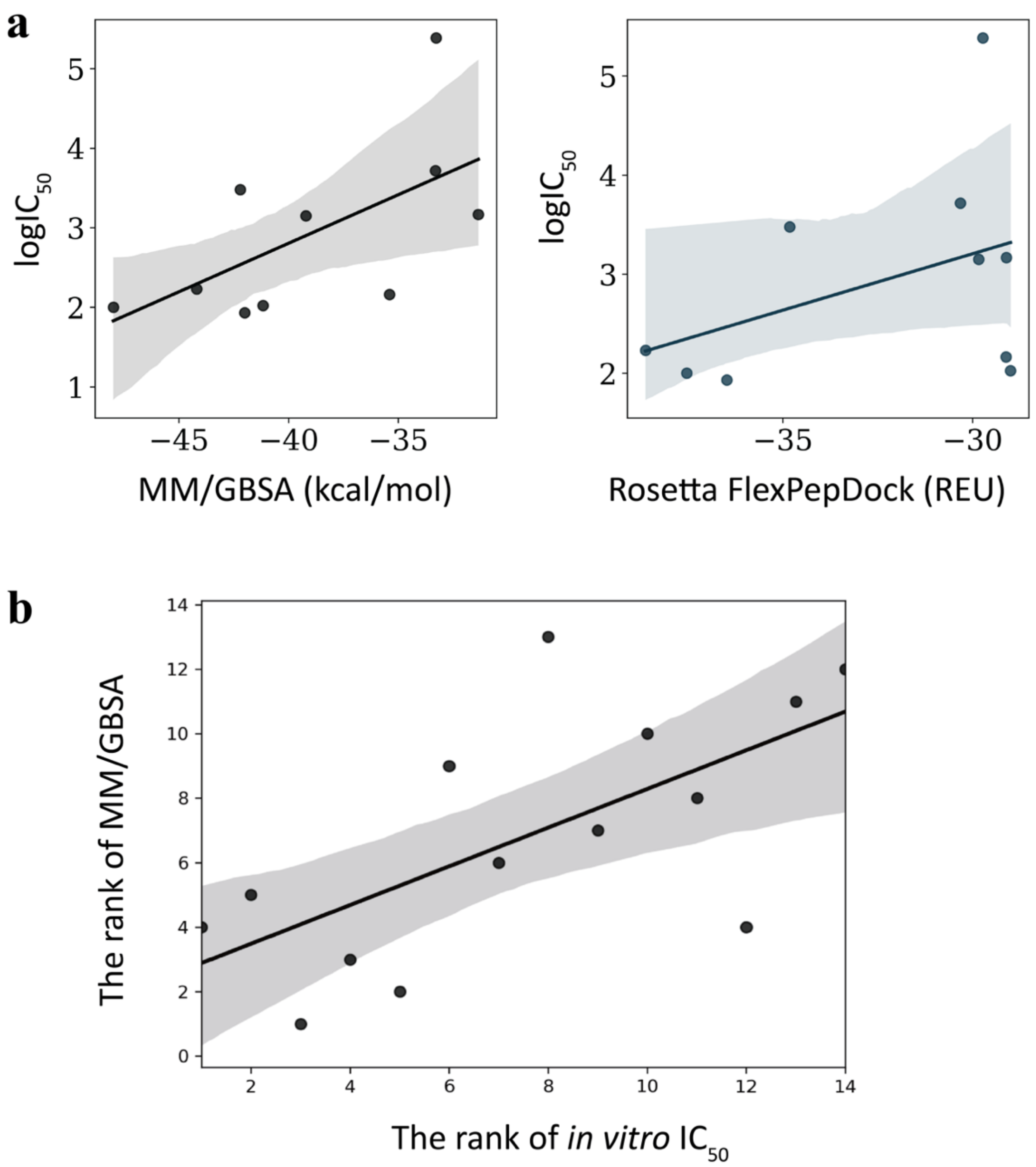

Supplementary Figure 2: a) Correlation plots of MM/GBSA vs. Log(IC_{50}) (left) and Rosetta FlexPepDock scores vs. Log(IC_{50}) (right) of 10 experimentally tested peptides comprising only natural amino acids. b) Correlation plot of MM/GBSA and IC_{50} rankings of 14 experimentally tested peptides (r^2 = 0.6). Source data are provided as a Source Data file.

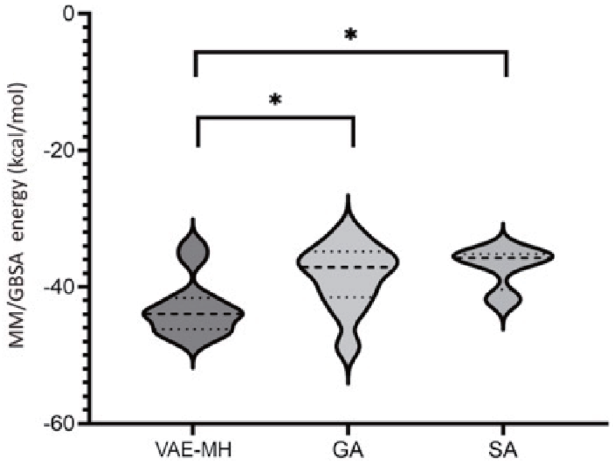

Supplementary Figure 3: Violin plots of MM/GBSA binding free energies for the top 30 peptides obtained from VAE-MH 2, GA optimization, and SA optimization, respectively. The shaded area represents uncertainty; * indicates a p-value of 0.01. Source data are provided as a Source Data file.

a

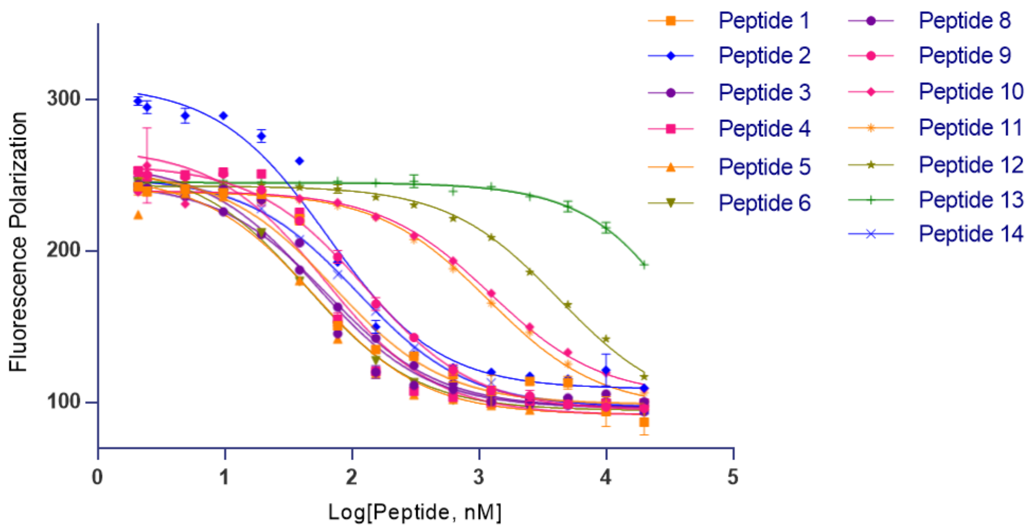

b

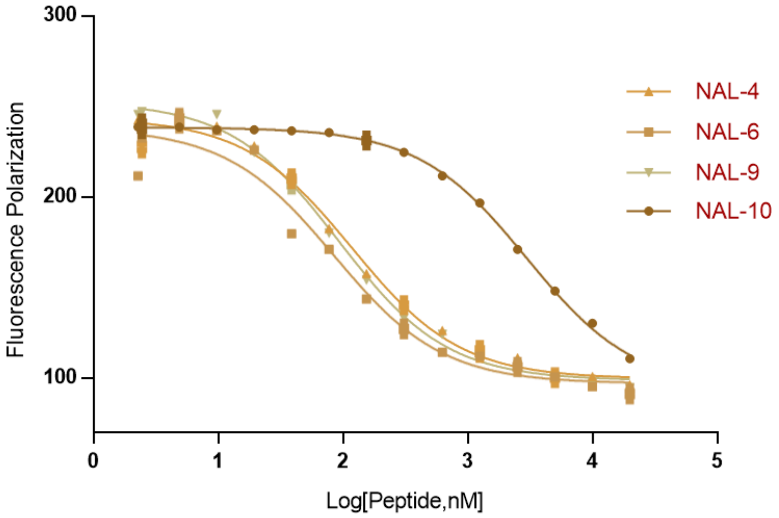

300
200
100
0
1
2
3
4
5
Fluorescence Polarization
Log[Peptide,nM]
NAL-4
NAL-6
NAL-9
NAL-10

c

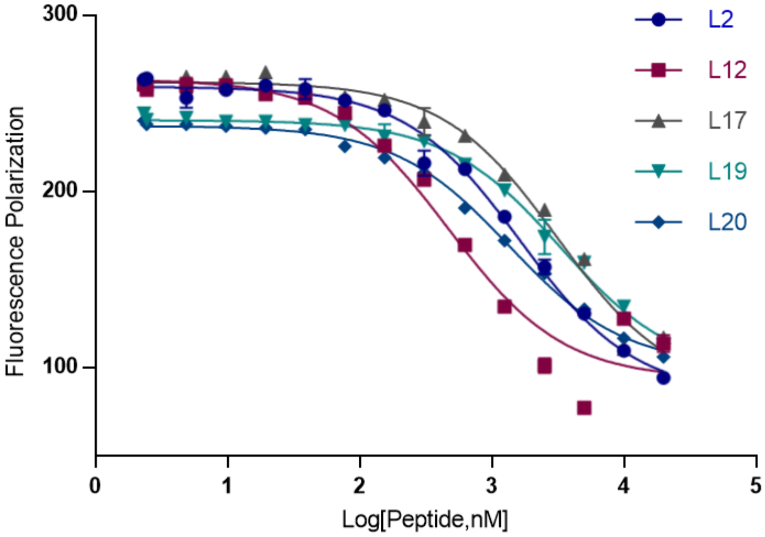

300
200
100
0
1
2
3
4
5
Fluorescence Polarization
Log[Peptide,nM]
L2
L12
L17
L19
L20

d

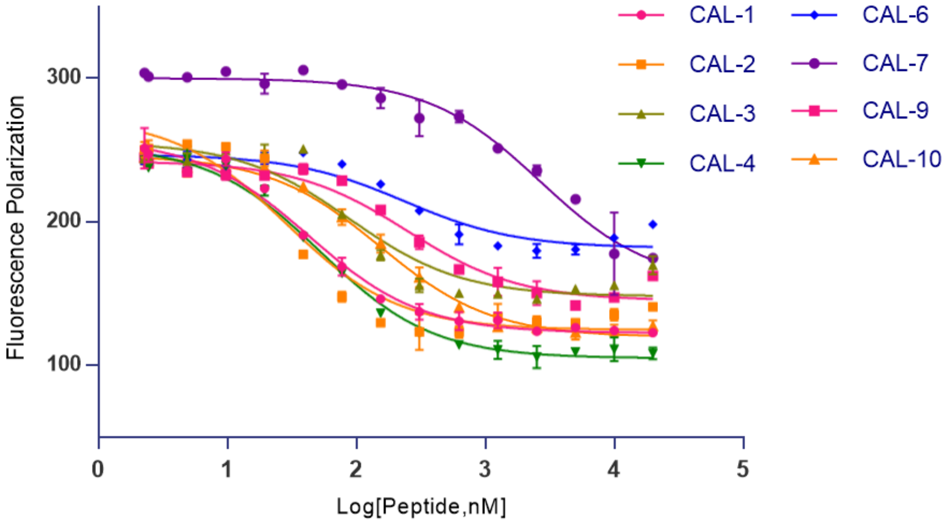

Supplementary Figure 4: Competition for binding to β-catenin by various peptides as monitored by FP assay. Each reaction contained 10 nM fluorescein-labeled peptide fAx6-2 as probe, 50 nM GST-β-catenin, and increasing concentrations of competing peptide in 20 mM Tris, pH 8.8, 300 mM NaCl, 0.01% Triton-X100. (a) Peptides 1-14; (b) peptides NAL-4, -6, -9, and -10; (c) peptides L2, L12, L17, L19, and L20; and (d) peptides CAL-1, -2, -3, -4, -6, -7, -9, and -10. Data shown represent the mean ± SD of three independent experiments. Source data are provided as a Source Data file.

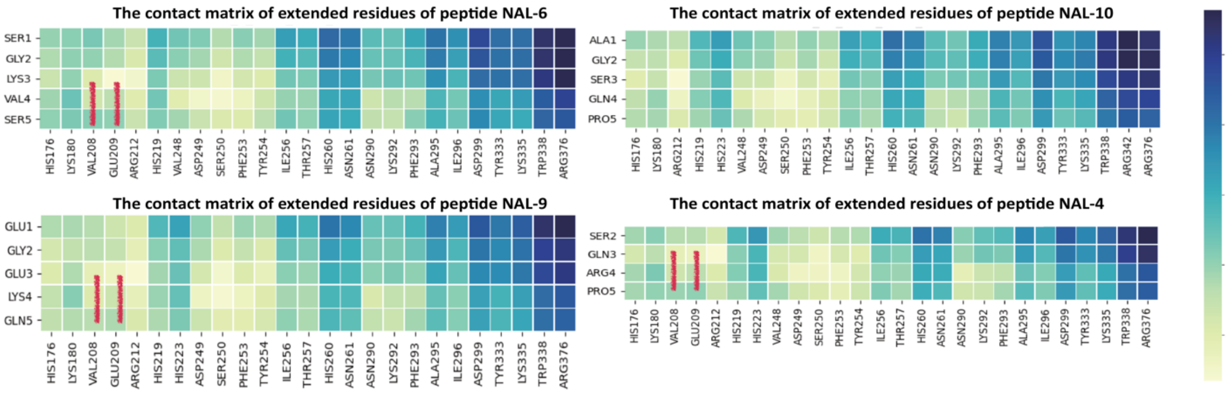

Supplementary Figure 5: Residue contact maps of β-catenin and the four N-terminally extended peptides. Residues on horizontal axis belong to β-catenin and on vertical axis belong to the peptides. The scale bar represents the distance (in Å) between each contact pair. Source data are provided as a Source Data file.

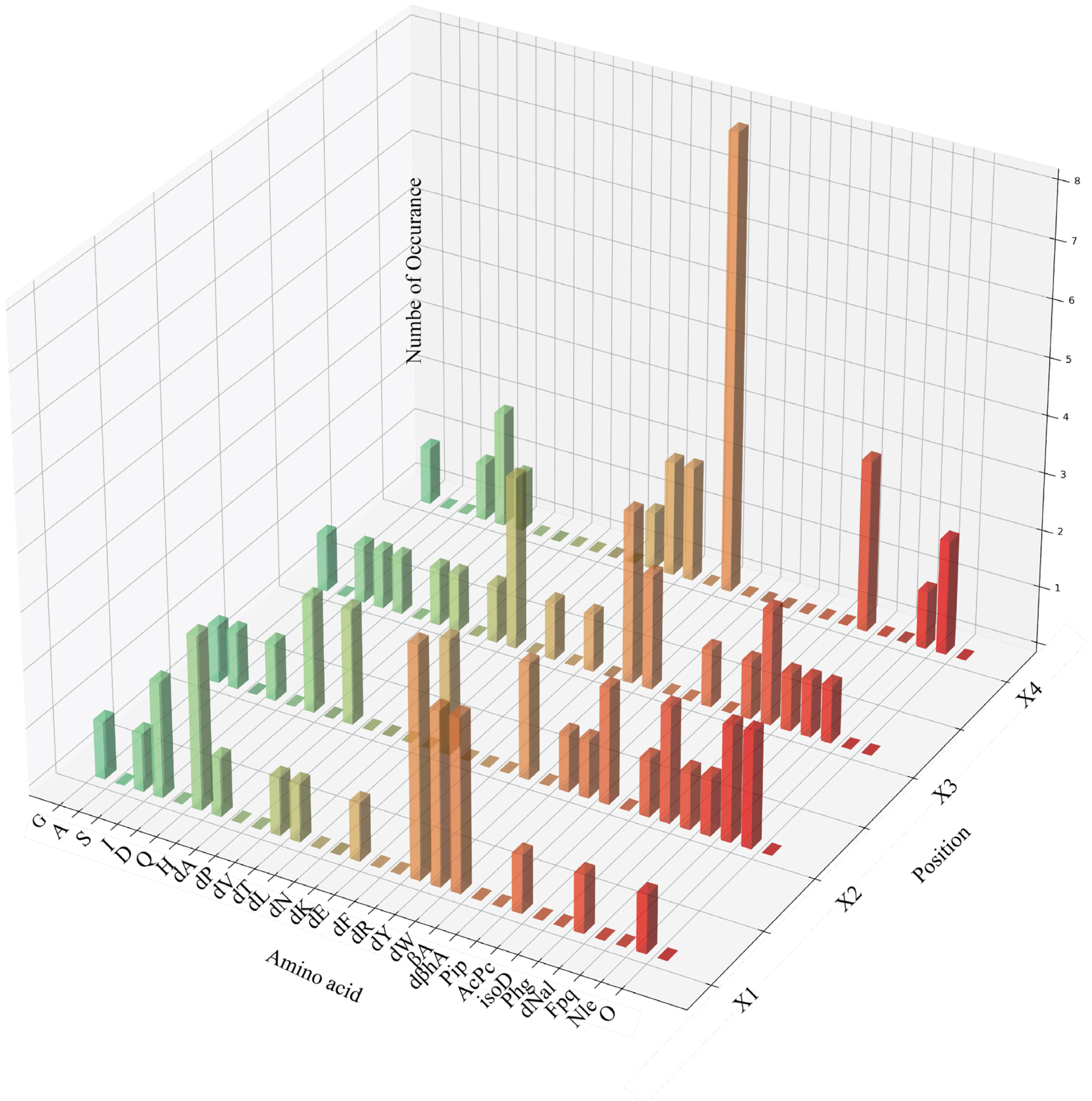

Supplementary Figure 6: Histogram showing the sequence specificity of β-catenin at the N-terminal extension to the core sequence YPEDILDKHLQRV (positions X1-X4), as displayed by the number of OBOC library-selected sequences (z axis) containing a particular amino acid (y axis) at a given peptide position (x axis). Source data are provided as a Source Data file.

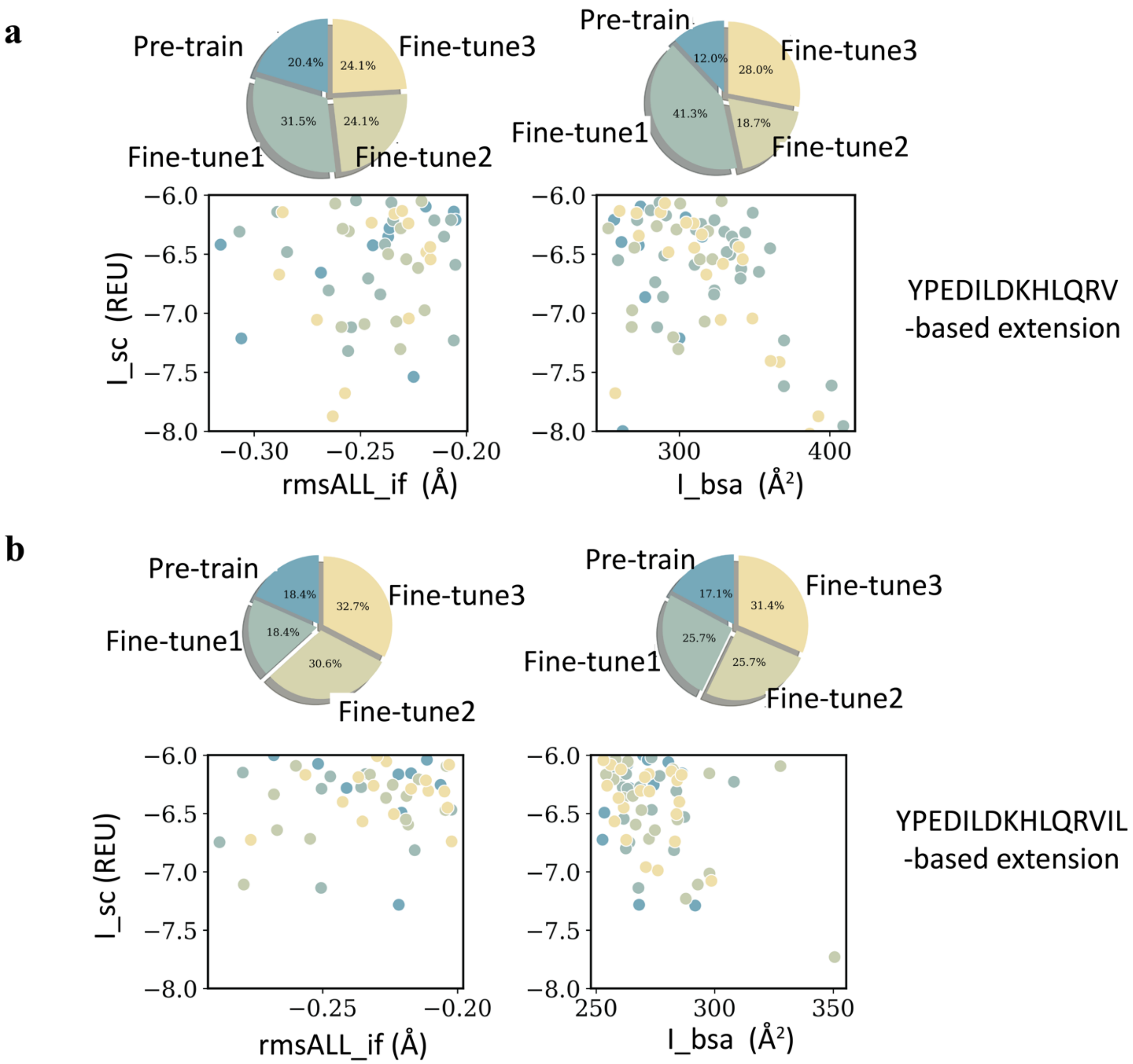

Supplementary Figure 7: a) Enrichment analysis for YPEDILDKHLQRV-based C-terminal extension. Pie chart showing the percentages of peptides selected for MM/GBSA evaluation that satisfy the cutoff criteria (rmsALL_if <-0.2 Å; I_sc < -6 REU; I_bsa >250 Å^2) for each of the four AI models. Scatter plots of I_sc vs. rmsALL_if (left) and I_sc vs. I_bsa (right) for peptides drawn from the above pie charts. b) Enrichment analysis for YPEDILDKHLQRVIL-based C-terminal extension. Pie chart showing the percentages of peptides selected for MM/GBSA evaluation that satisfy the cutoff criteria (rmsALL_if <-0.2 Å; I_sc < -6 REU; I_bsa >250 Å^2) for each of the four AI models. Scatter plots of I_sc vs. rmsALL_if (left) and I_sc vs. I_bsa (right) for peptides drawn from the above pie charts. Different colors correspond to peptides generated by different AI models. Source data are provided as a Source Data file.

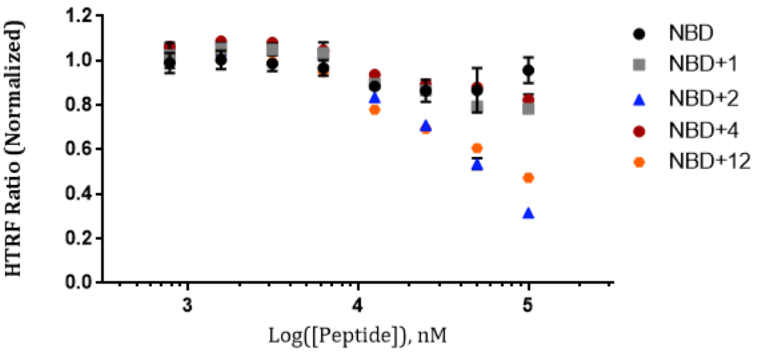

Supplementary Figure 8: Competition for binding to NEMO by the four AI-generated peptides compared to the parent peptide NBD, as monitored by competitive homogenous time-resolved fluorescence (HTRF) assays. Source data are provided as a Source Data file.

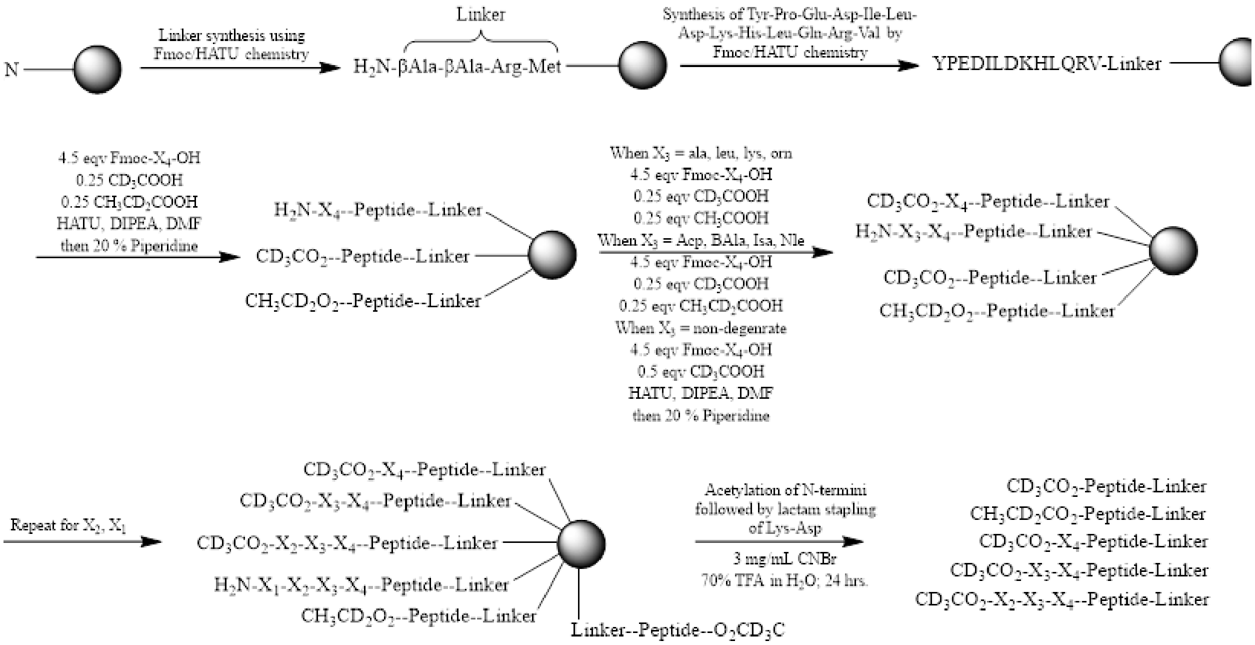

Supplementary Figure 9: Scheme showing the synthesis of the OBOC peptide library, which contains a tetrapeptide extension (X_1–X_4) to the N-terminus of core peptide YPE<u>D</u>ILD<u>K</u>HLQRV (where the underlined residues are stapled by the formation of an intramolecular amide bond).

Peptide ID	Purity (%)	Calculated [M+H]+	Observed [M+H]+
**1**	95.5	2074.14	2074.21
**2**	97.7	2076.10	2076.12
**3**	97.9	2482.25	2482.19
**4**	98.9	2087.10	2087.13
**5**	96.4	2093.06	2093.34
**7**	97.8	2482.25	2482.21
**8**	98.9	1832.99	1833.04
**9**	98.6	1989.08	1989.09
**10**	97.8	1876.01	1876.04
**11**	97.9	1762.92	1762.95
**12**	96.8	1785.94	1786.02
**13**	96.7	1771.92	1771.96
**14**	97.8	1832.99	1833.01
**NAL-4**	96.4	2301.29	2301.33
**NAL-6**	95.9	2291.29	2291.23
**NAL-9**	95.6	2404.29	2404.48
**NAL-10**	98.2	2273.24	2273.36
**L2**	96.7	2148.17	2148.21
**L12**	97	2162.14	2162.08
**L17**	96.9	2117.19	2117.13
**L19**	98.1	2146.18	2146.12
**L20**	96.5	2119.17	2119.25
**CAL-1**	96.3	2569.19	2569.21
**CAL-2**	97.1	2822.34	2822.42
**CAL-3**	97.3	3030.48	3030.53
**CAL-4**	97.5	2543.20	2543.27
**CAL-6**	97.2	2647.42	2647.51
**CAL-7**	96.7	2522.33	2522.38
**CAL-9**	95.5	2677.40	2677.69
**CAL-10**	97.1	2272.19	2272.48

Peptide 1

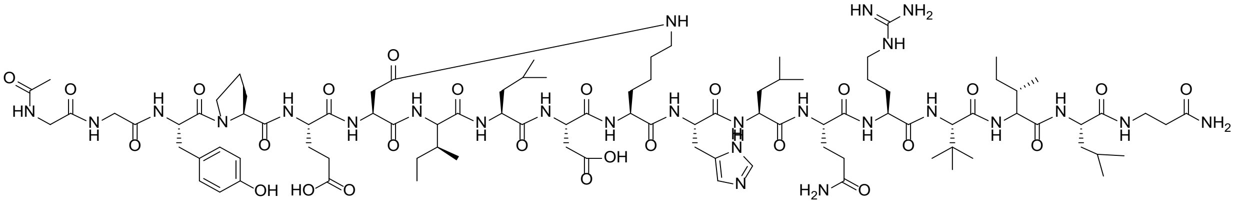

Exact Mass: 2073.1371

Purity assessment by UPLC (214 nm):

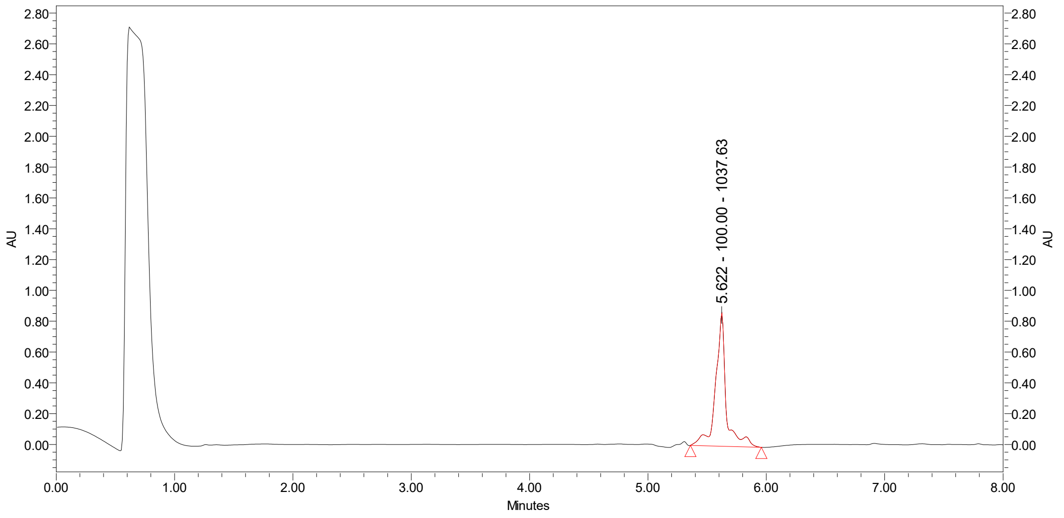

HR-MS:

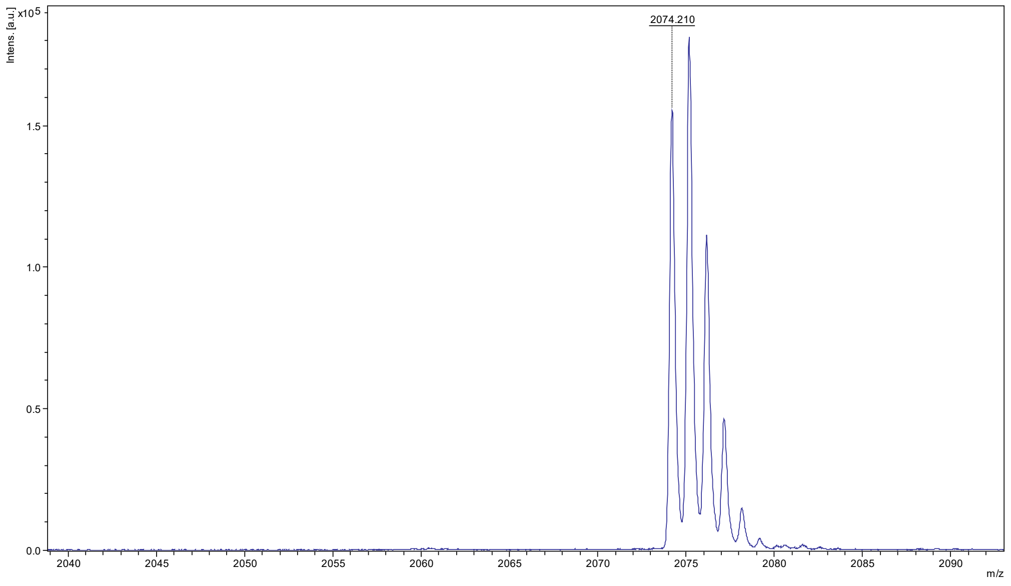

Peptide 2

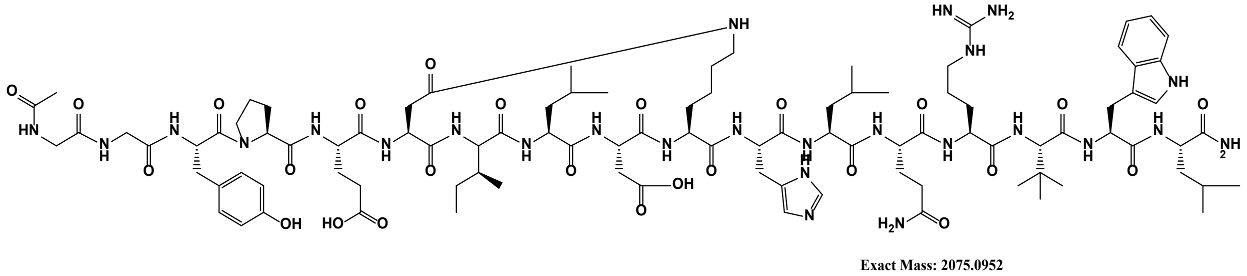

Purity assessment by UPLC (214 nm):

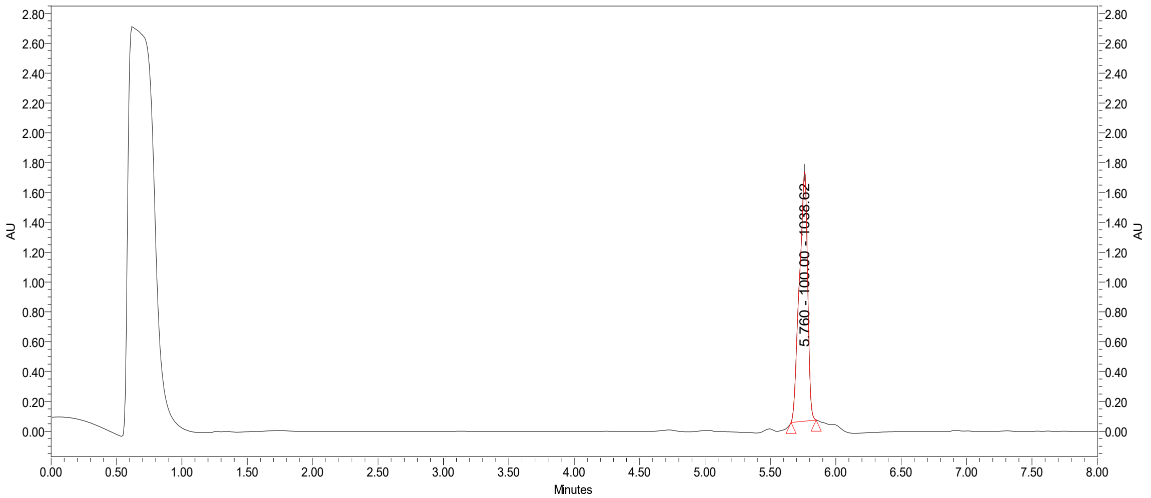

HR-MS:

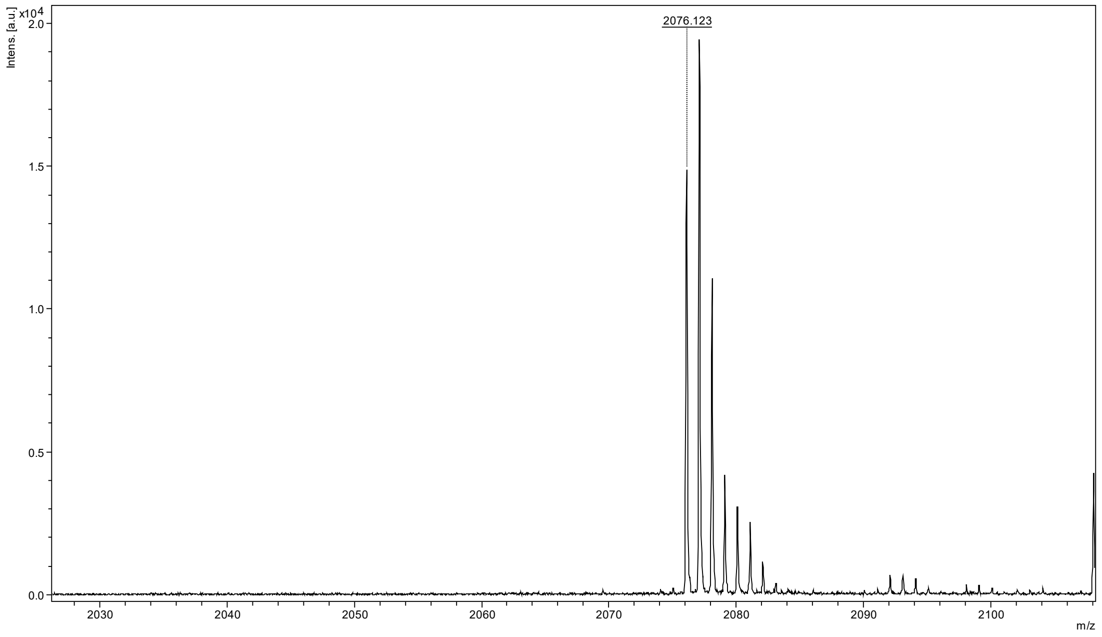

Peptide 3

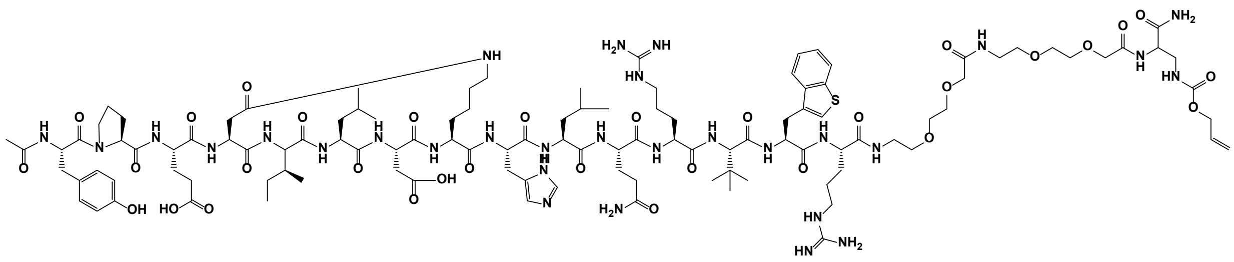

Exact Mass: 2481.2475

Purity assessment by UPLC (214 nm):

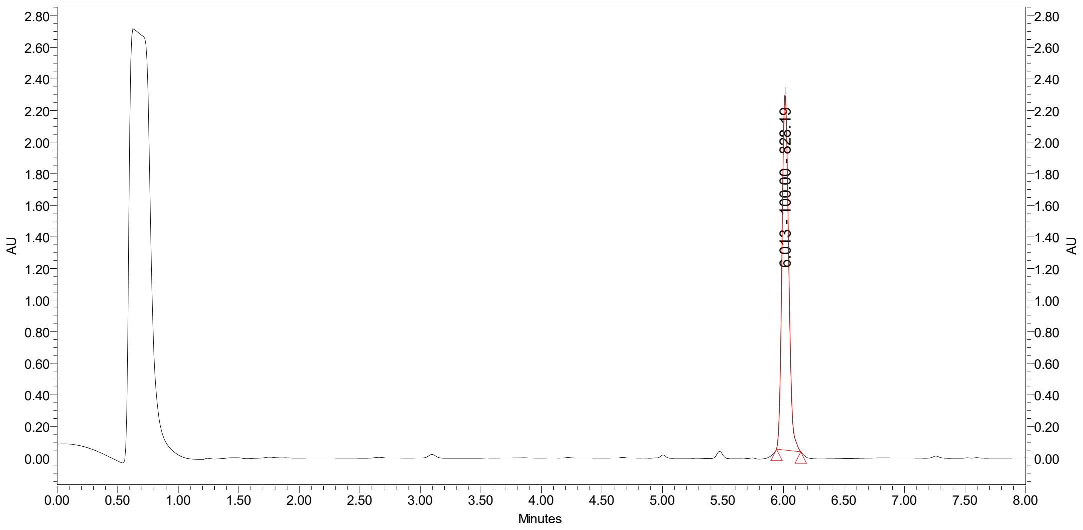

HR-MS:

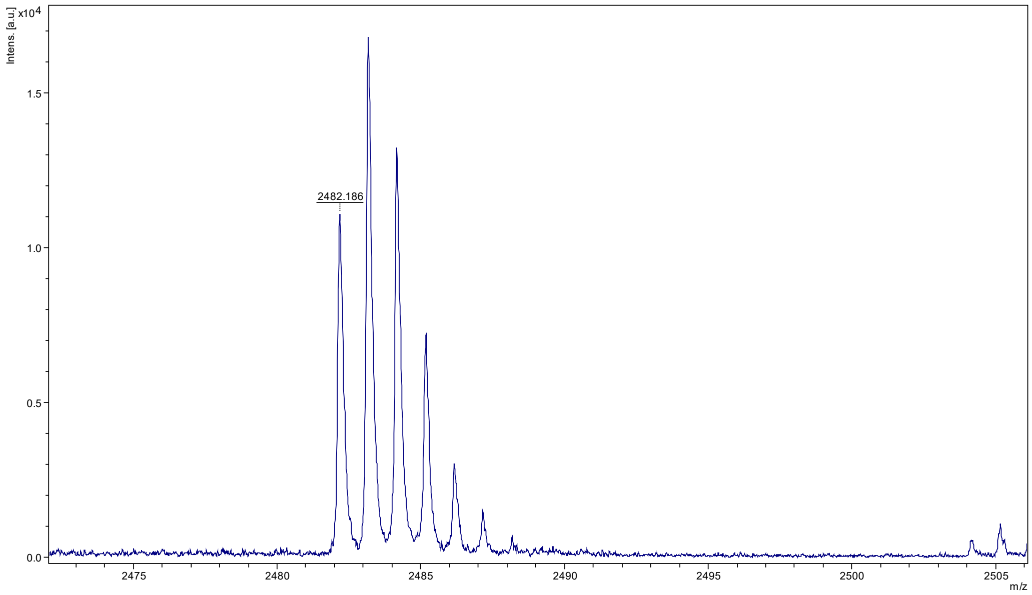

Peptide 4

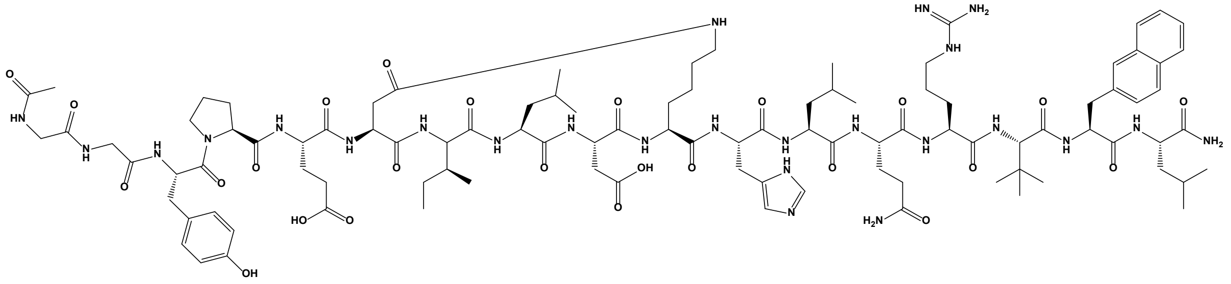

Exact Mass: 2086.1000

Purity assessment by UPLC (214 nm):

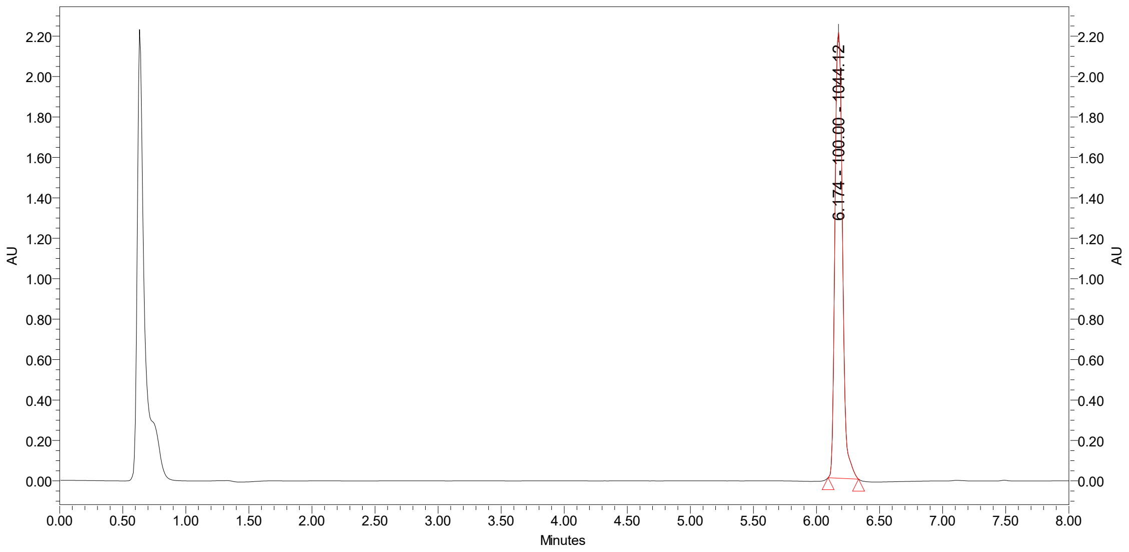

HR-MS:

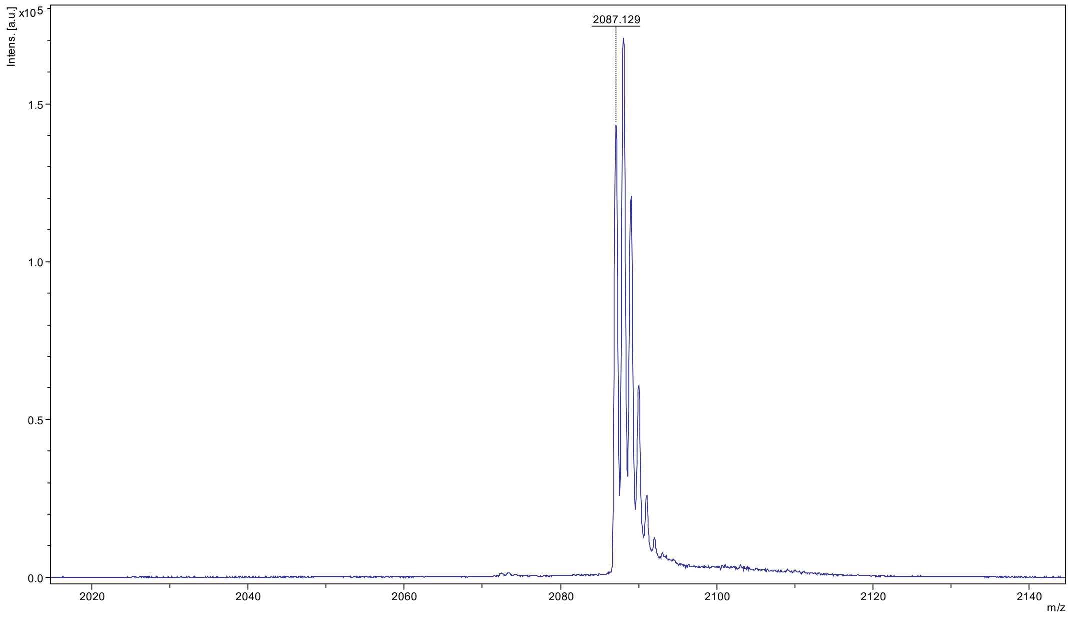

Peptide 5

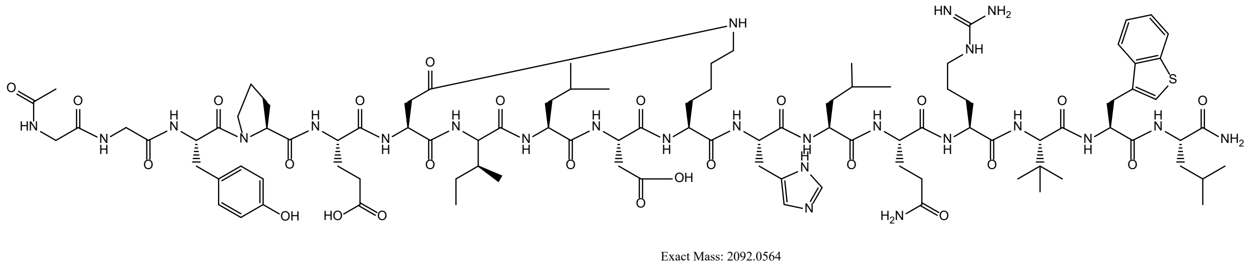

Purity assessment by UPLC (214 nm):

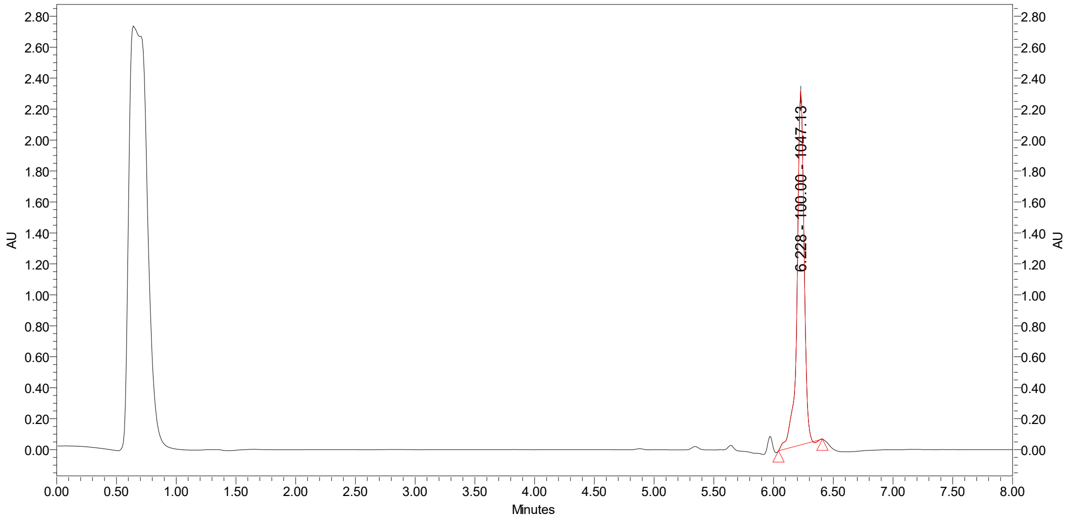

HR-MS:

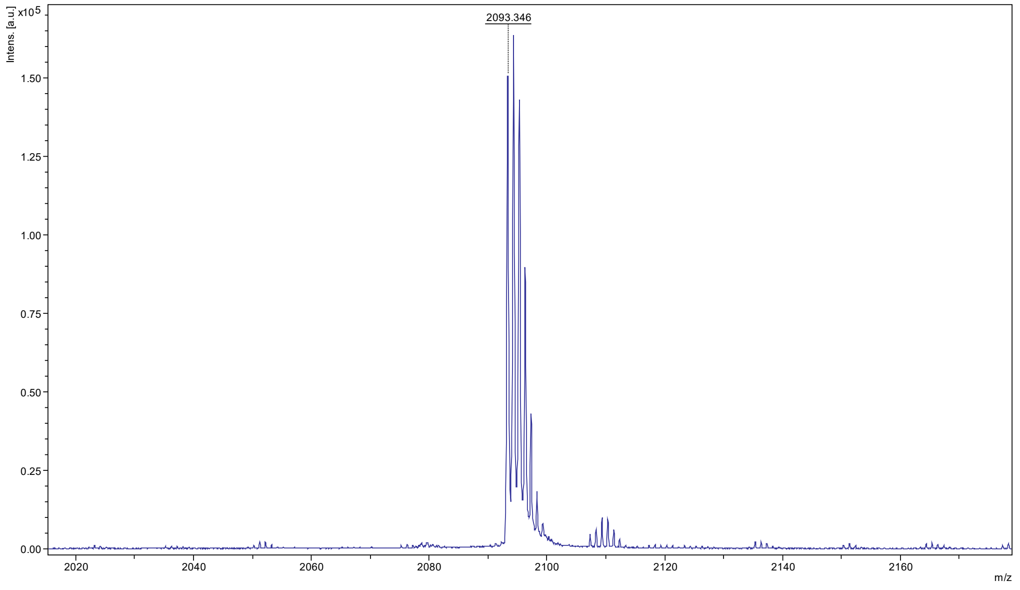

Peptide 7

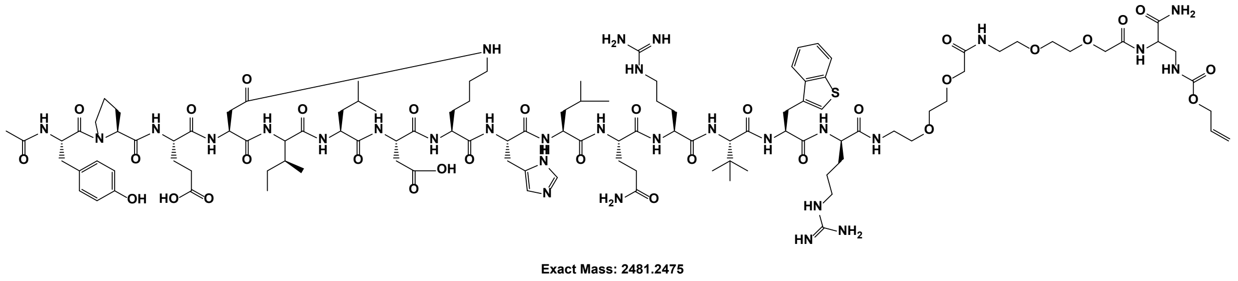

Purity assessment by UPLC (214 nm):

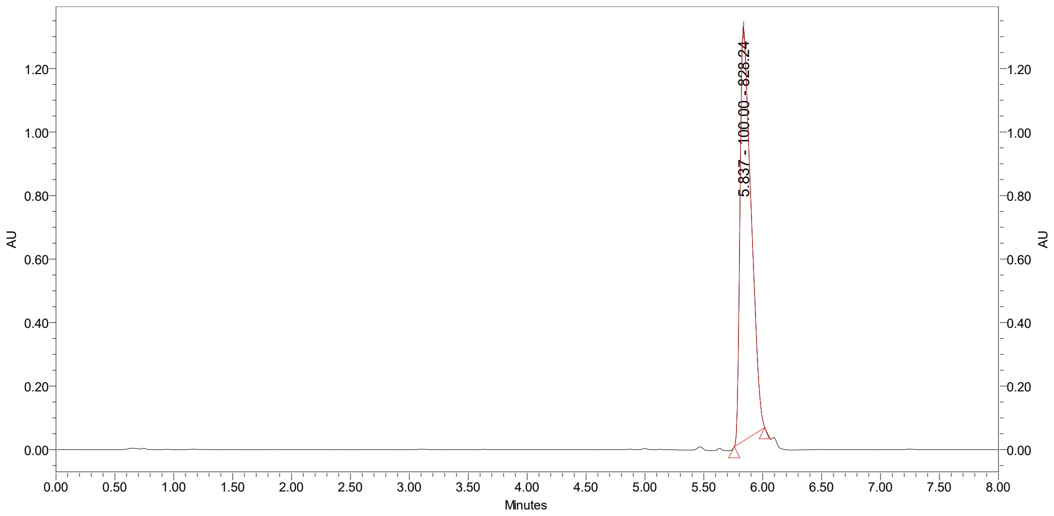

HR-MS:

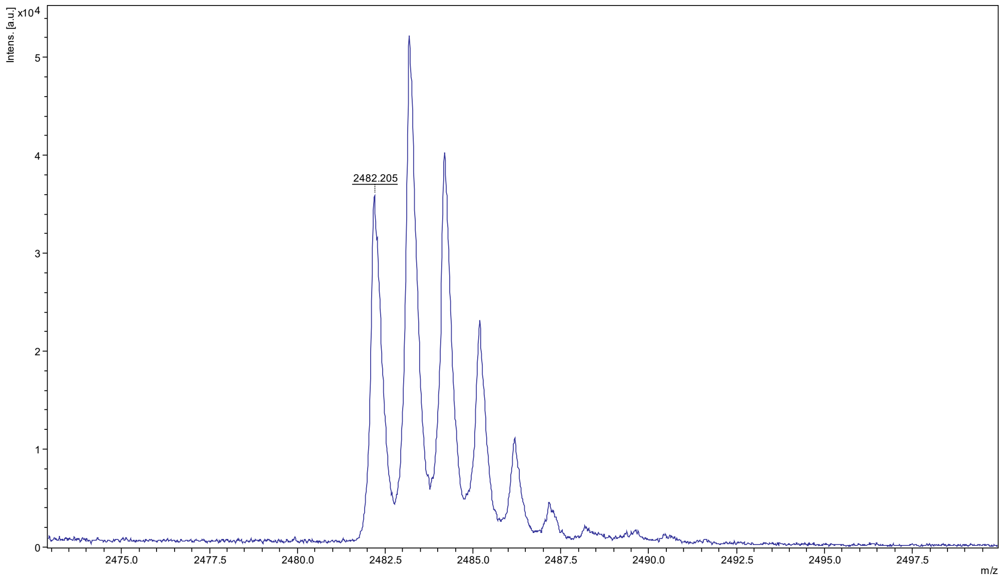

Peptide 8

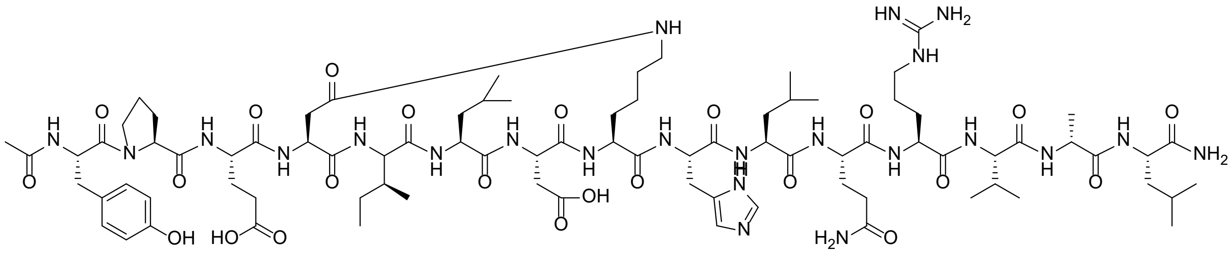

Exact Mass: 1831.9945

Purity assessment by UPLC (214 nm):

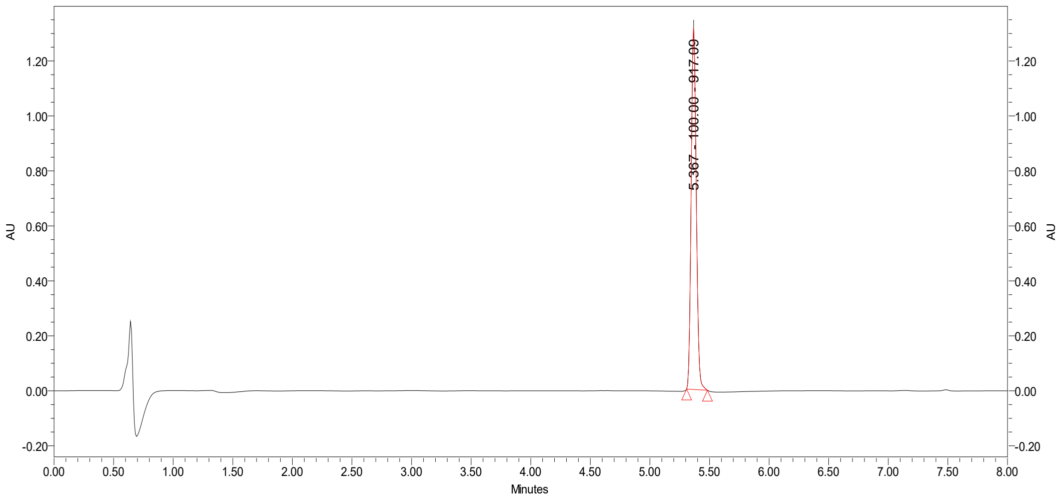

HR-MS:

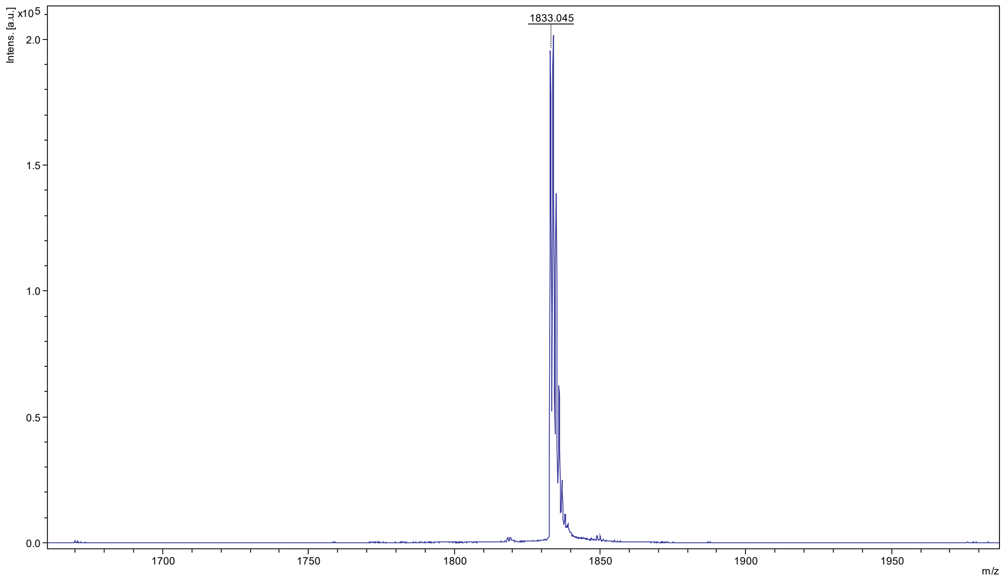

Peptide 9

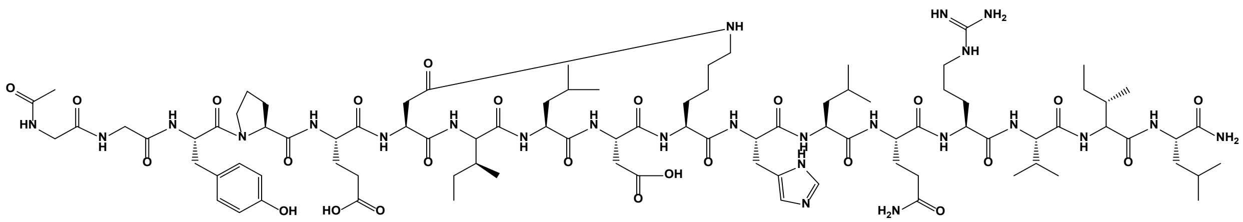

Purity assessment by UPLC (214 nm):

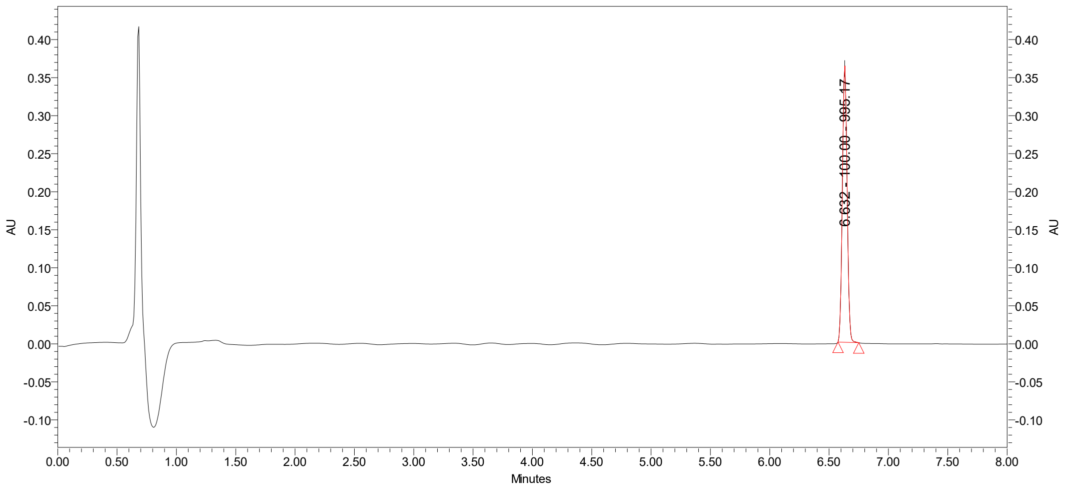

HR-MS:

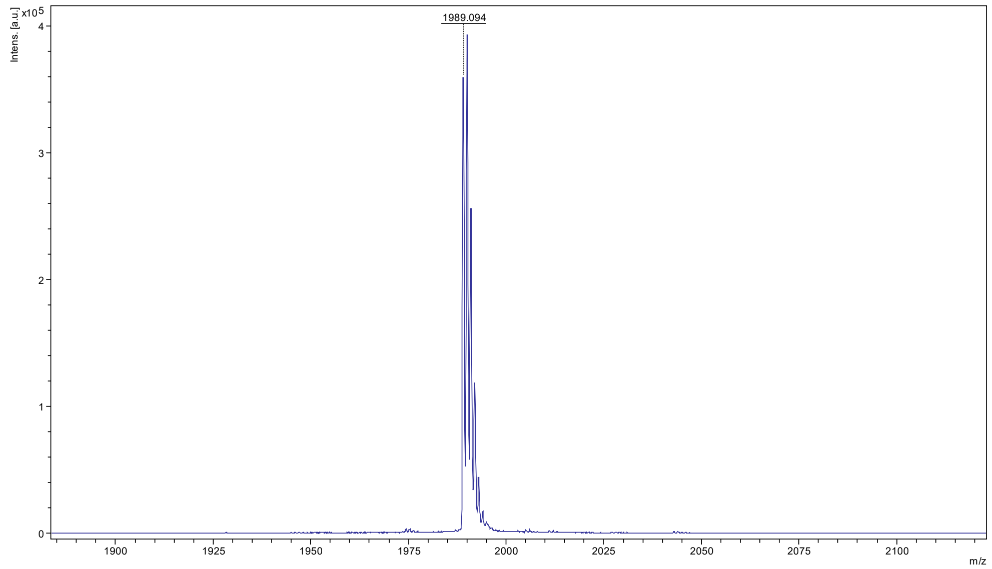

Peptide 10

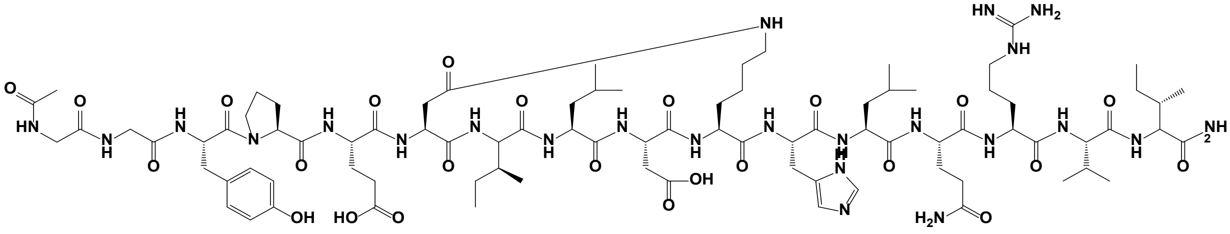

Purity assessment by UPLC (214 nm):

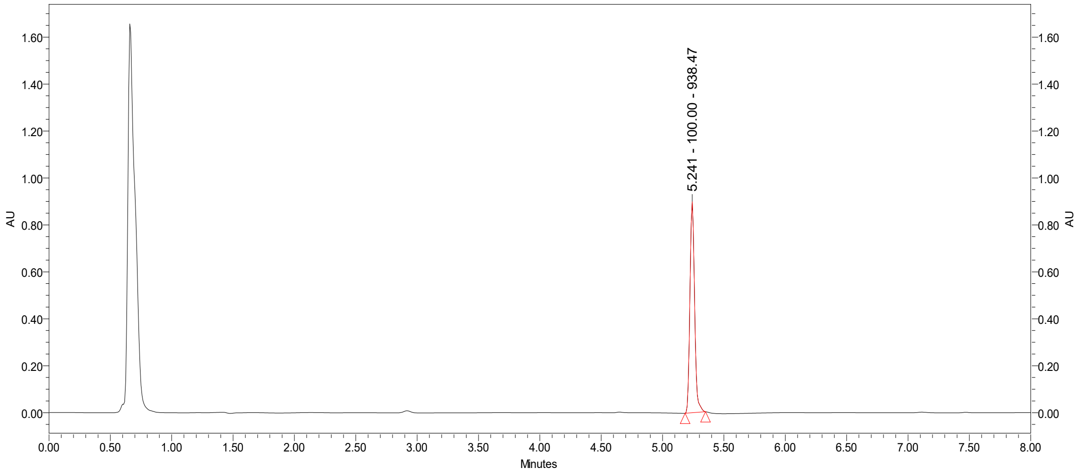

HR-MS:

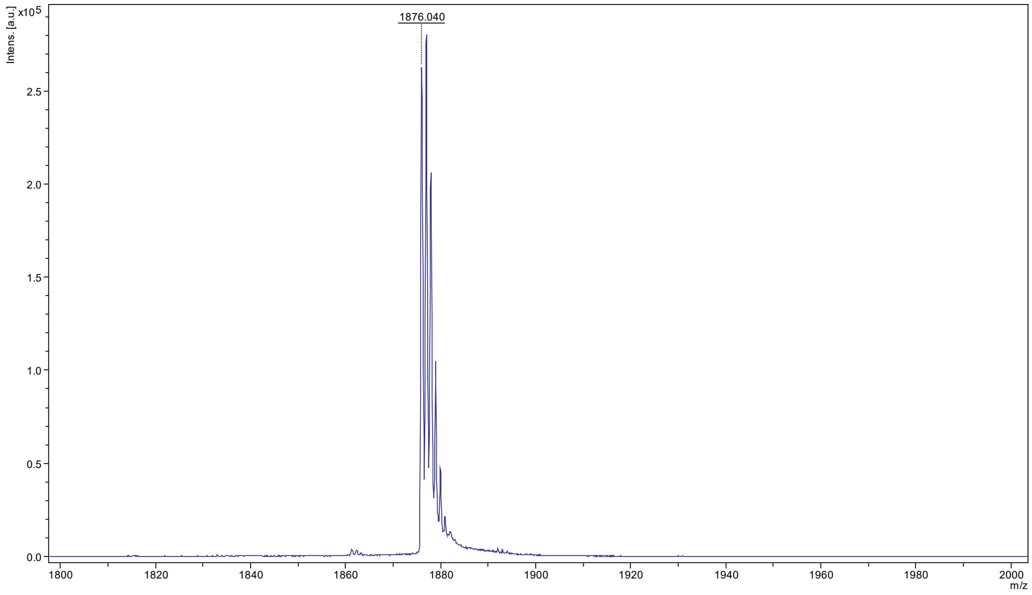

Peptide 11

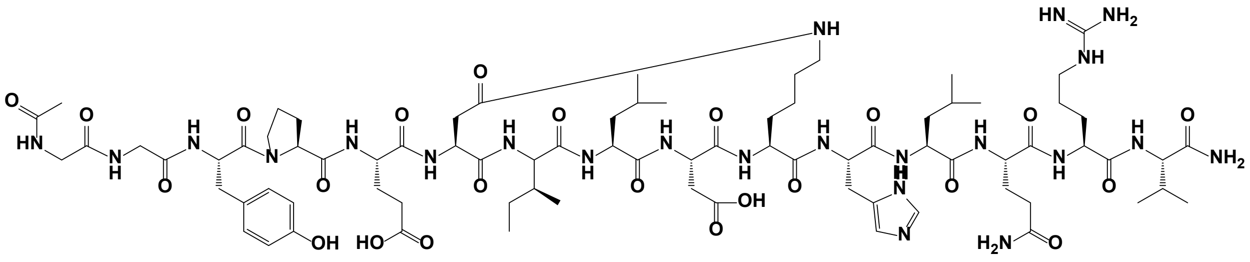

Exact Mass: 1761.9162

Purity assessment by UPLC (214 nm):

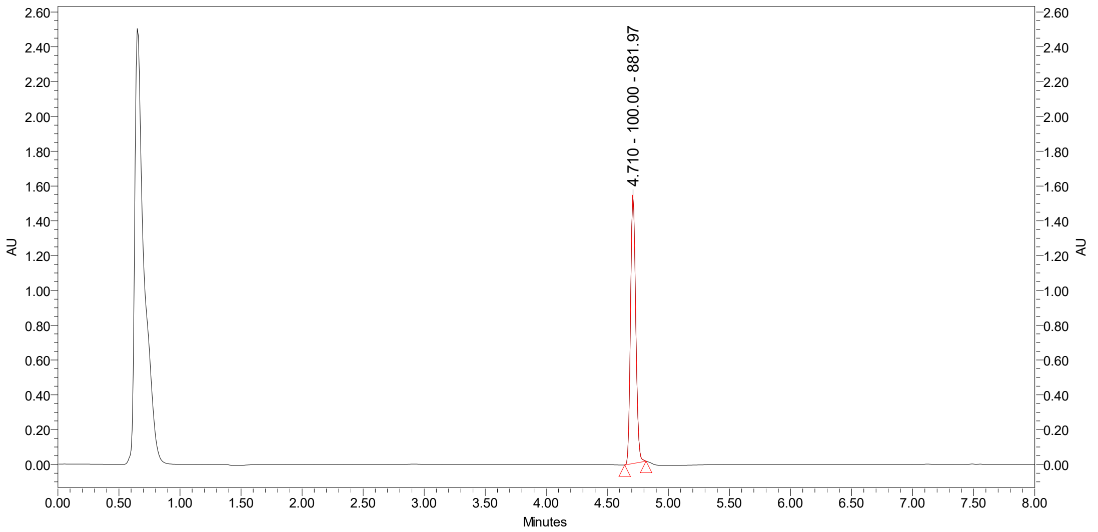

HR-MS:

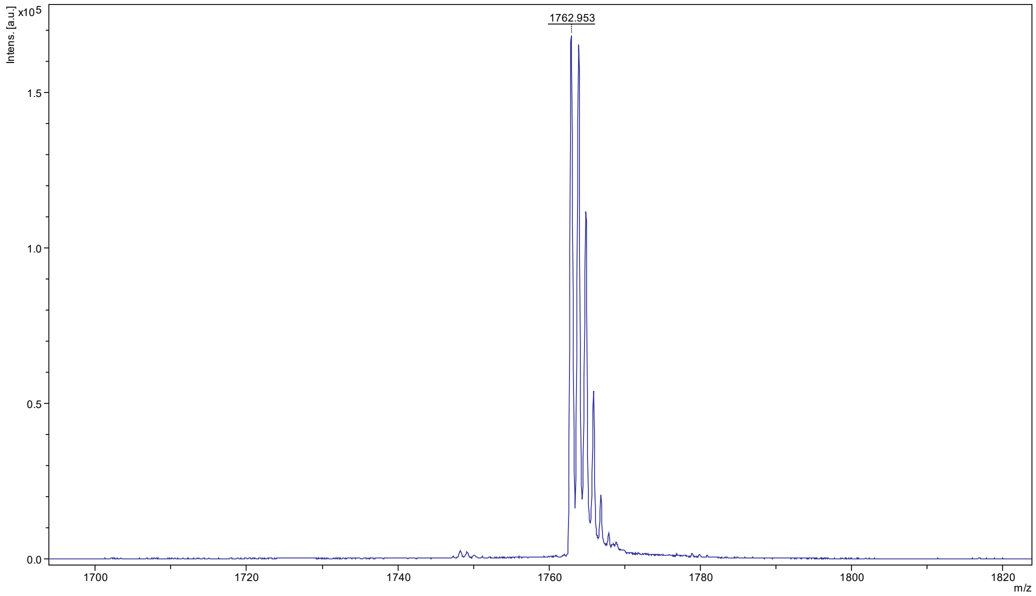

Peptide 12

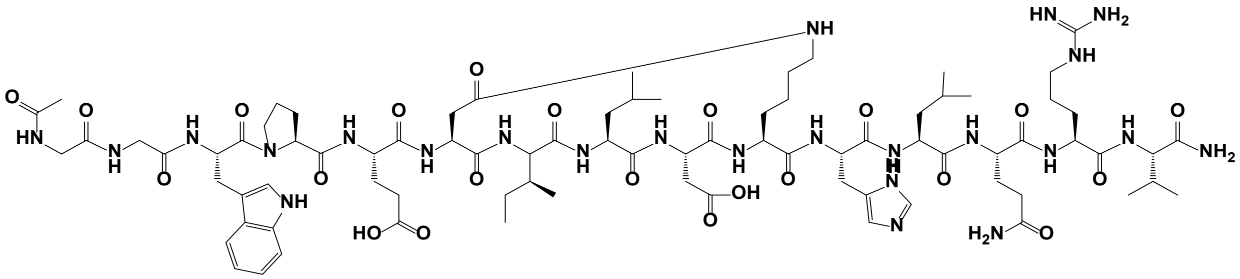

Exact Mass: 1784.9322

Purity assessment by UPLC (214 nm):

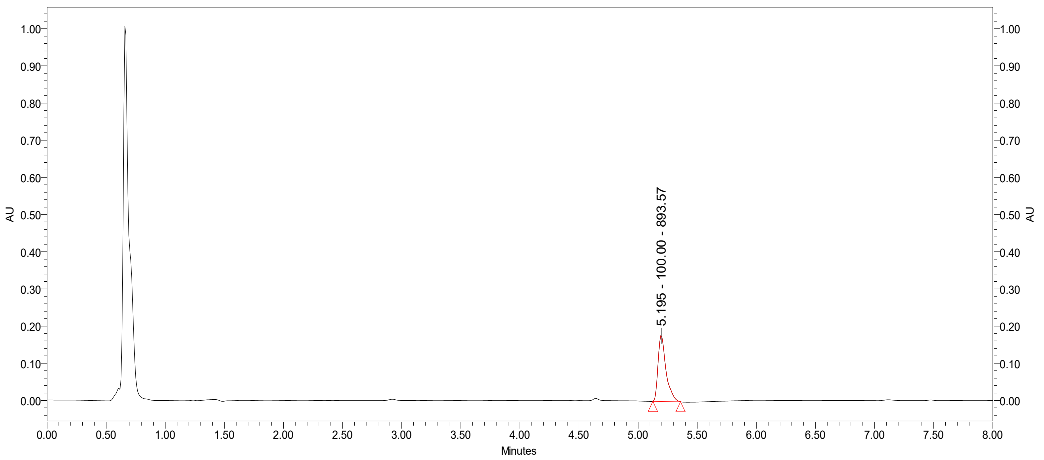

HR-MS:

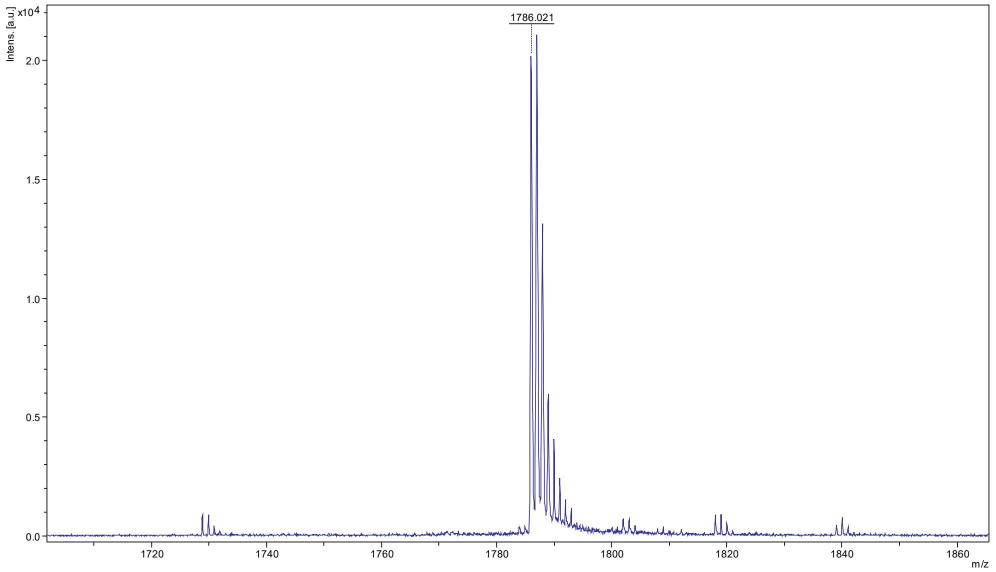

Peptide 13

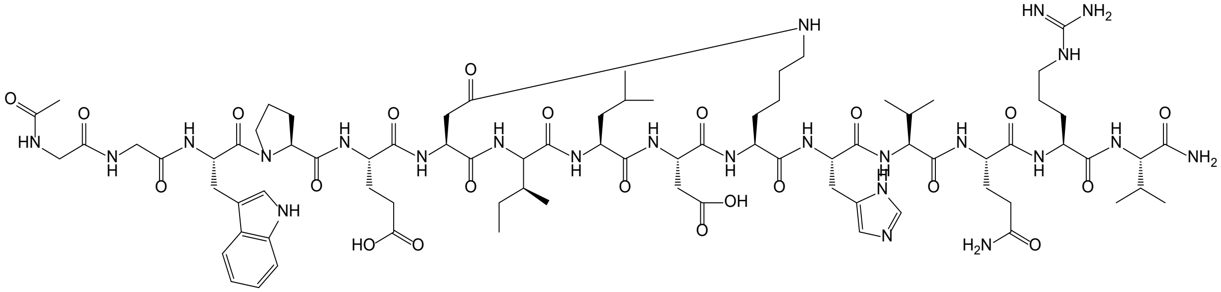

Exact Mass: 1770.9166

Purity assessment by UPLC (214 nm):

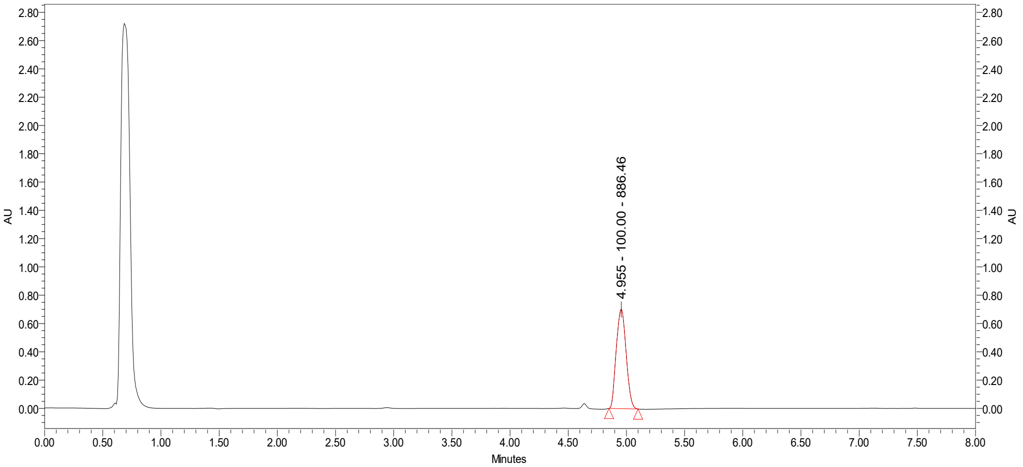

HR-MS:

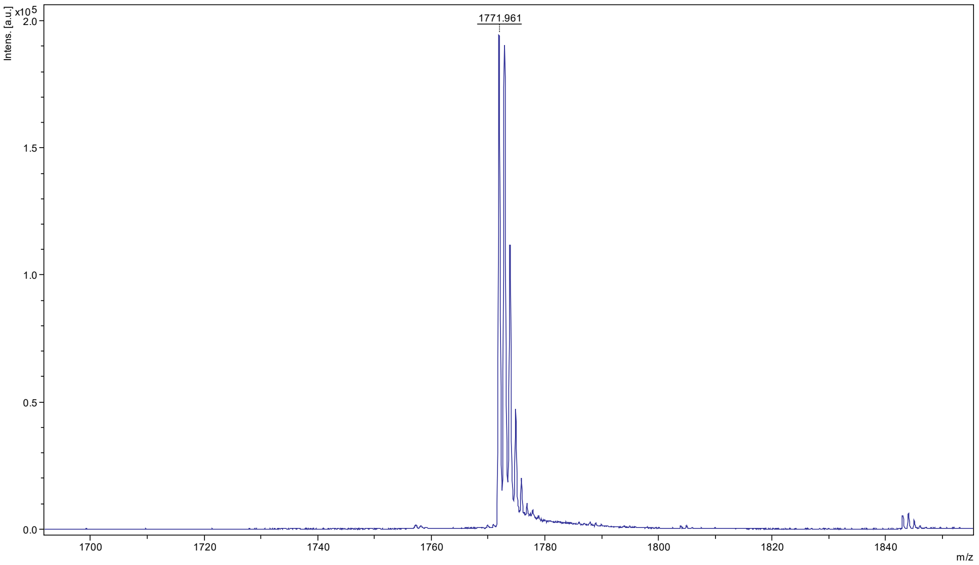

Peptide 14

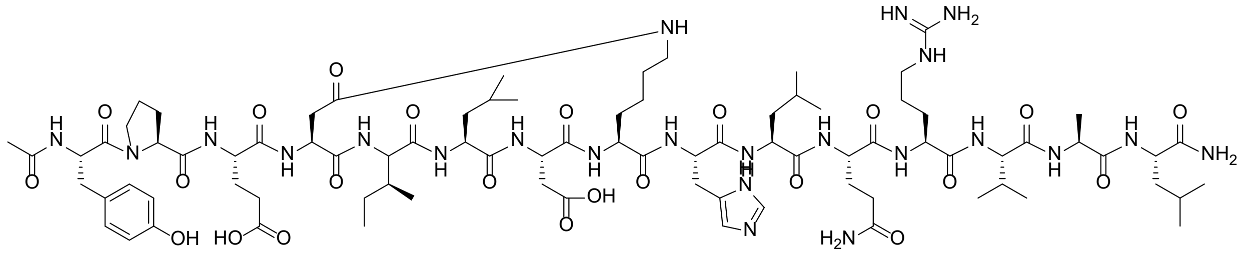

Purity assessment by UPLC (214 nm):

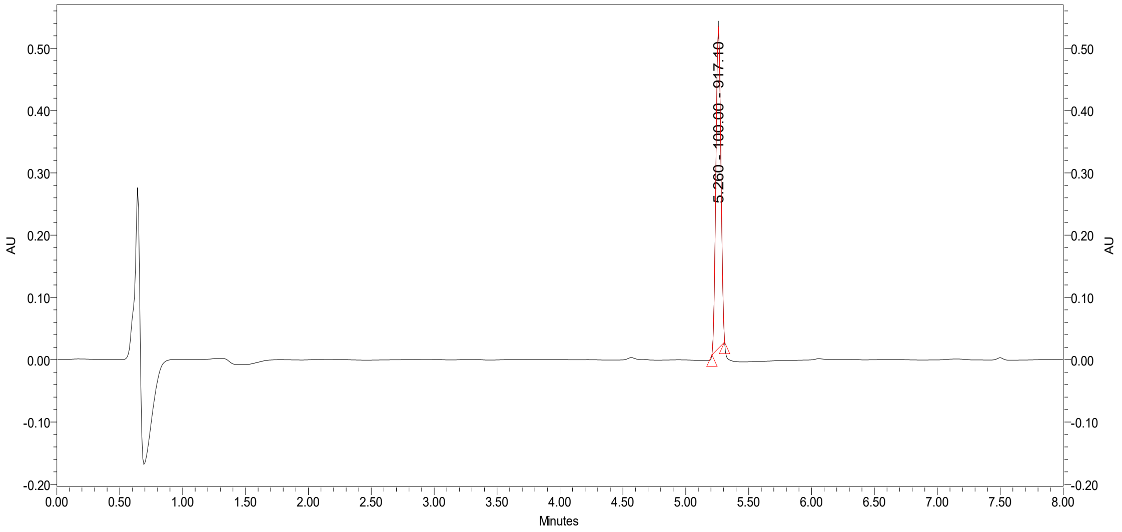

HR-MS:

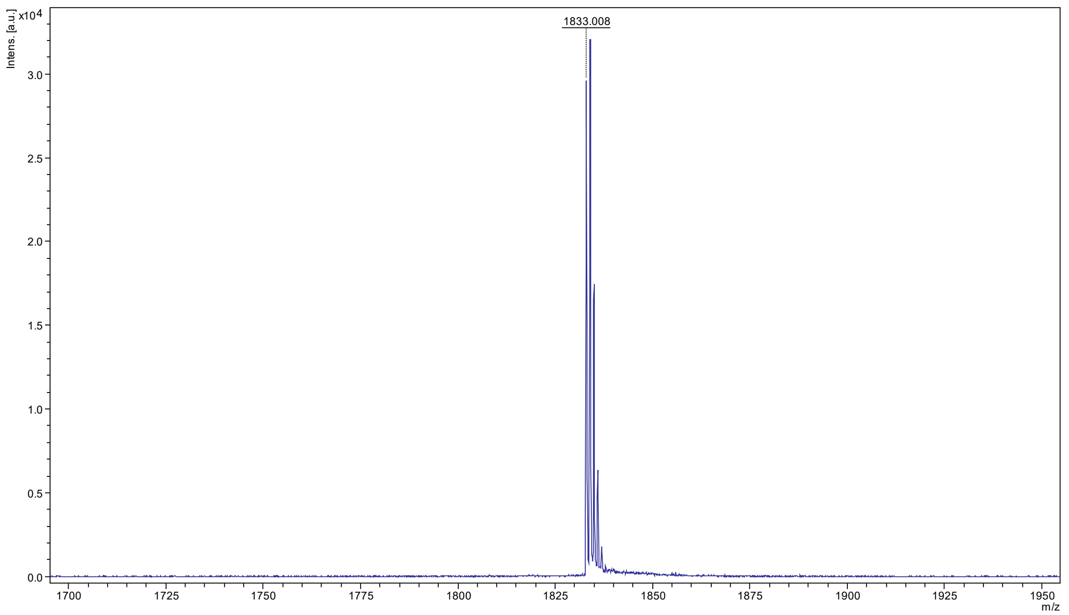

NAL-4

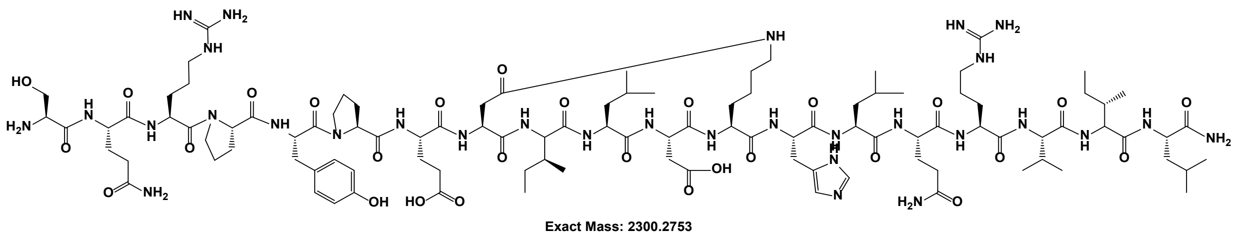

Purity assessment by UPLC (214 nm):

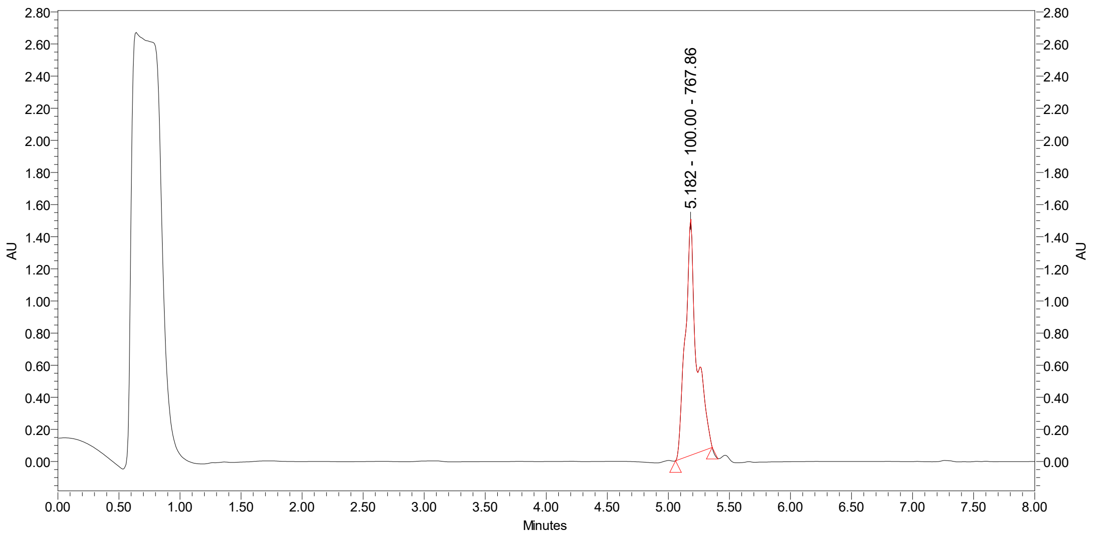

HR-MS:

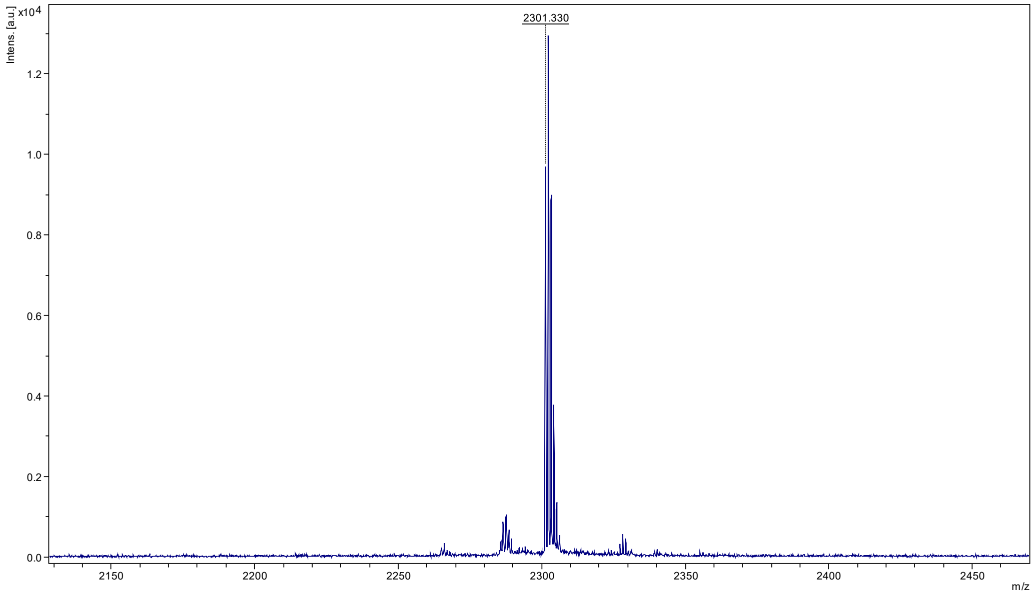

NAL-6

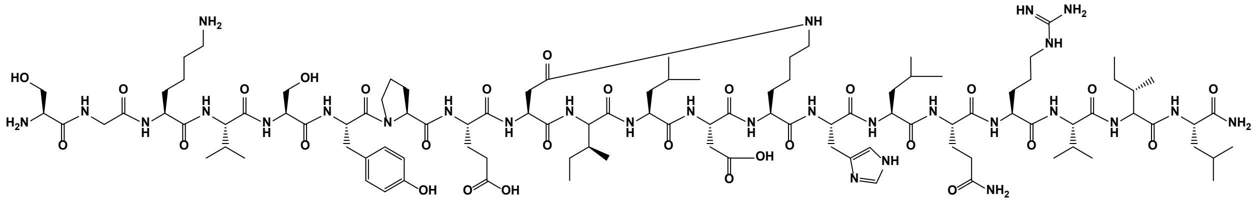

Purity assessment by UPLC (214 nm):

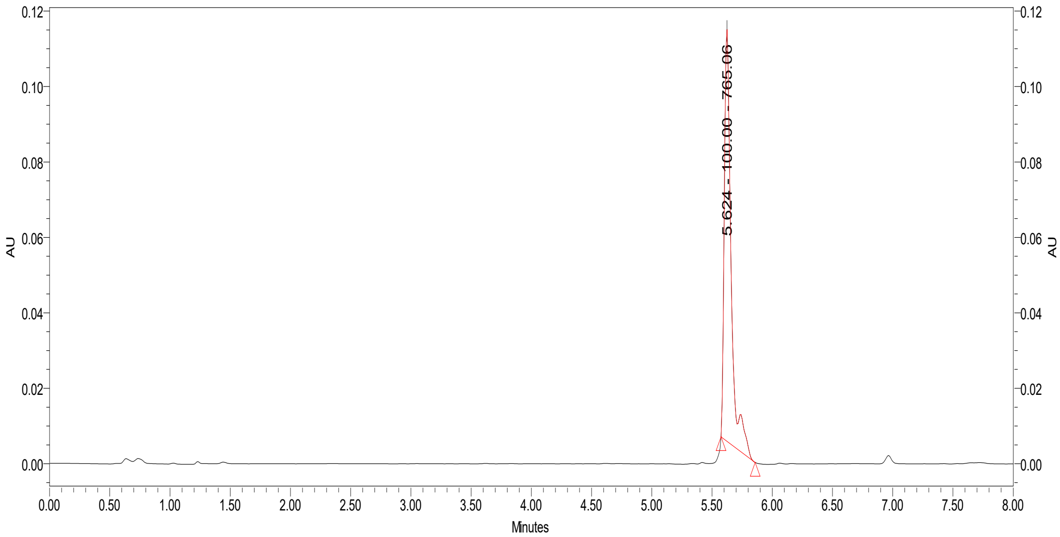

HR-MS:

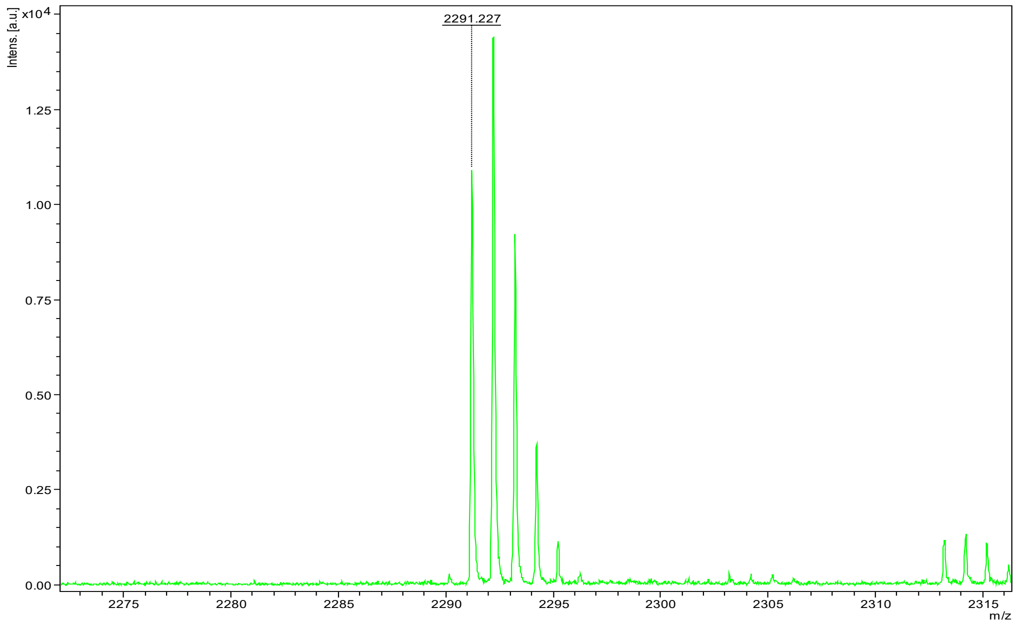

NAI-9

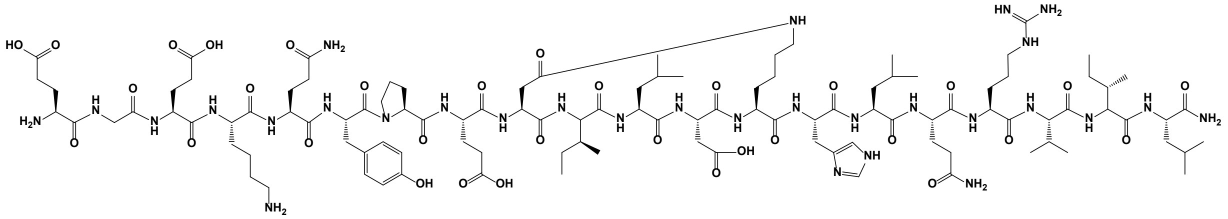

Purity assessment by UPLC (214 nm):

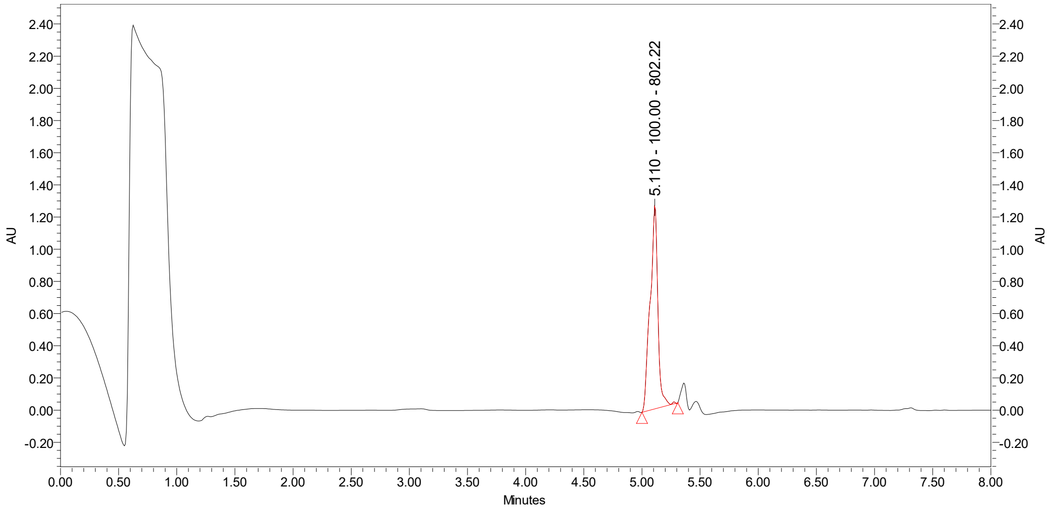

HR-MS:

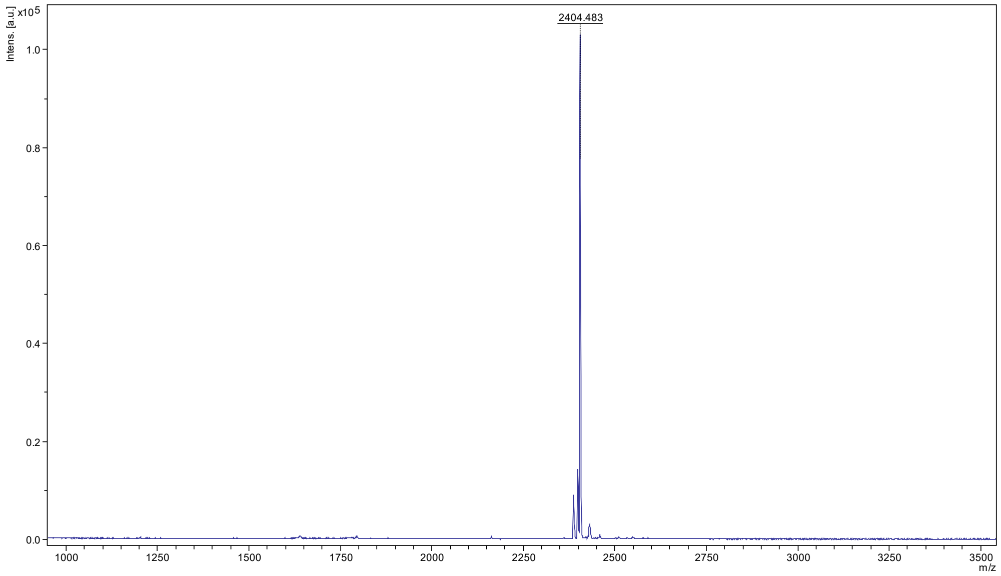

NAL-10

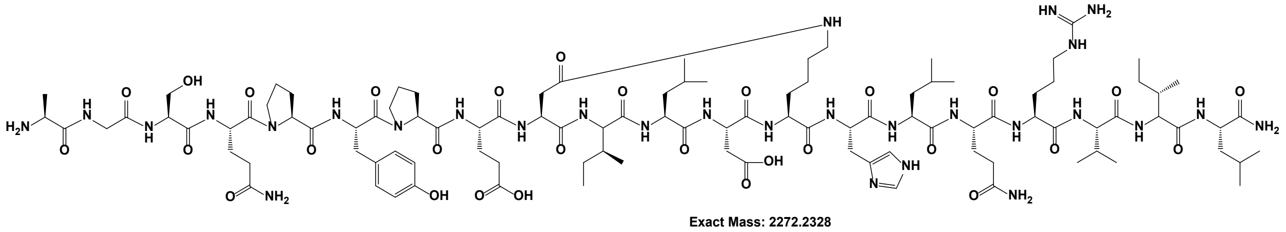

Purity assessment by UPLC (214 nm):

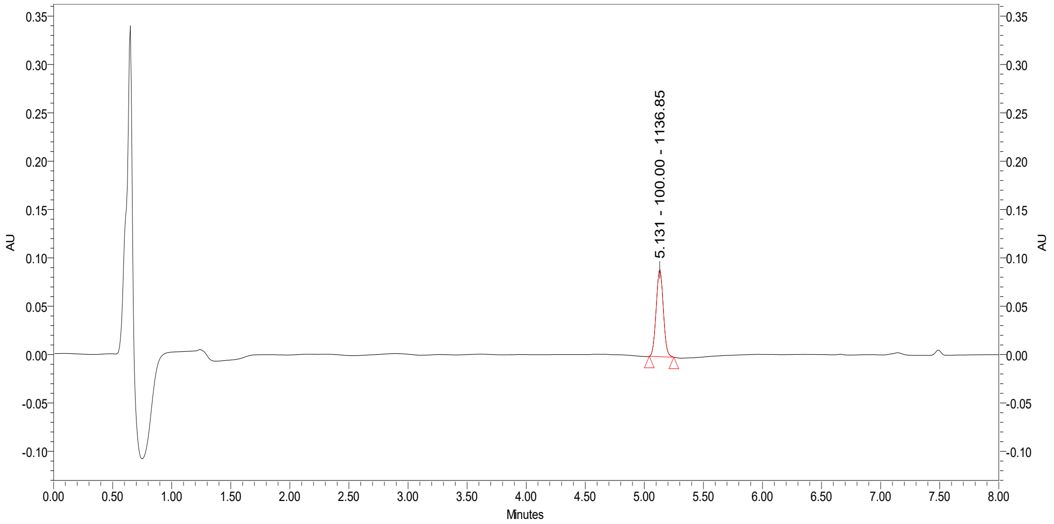

HR-MS:

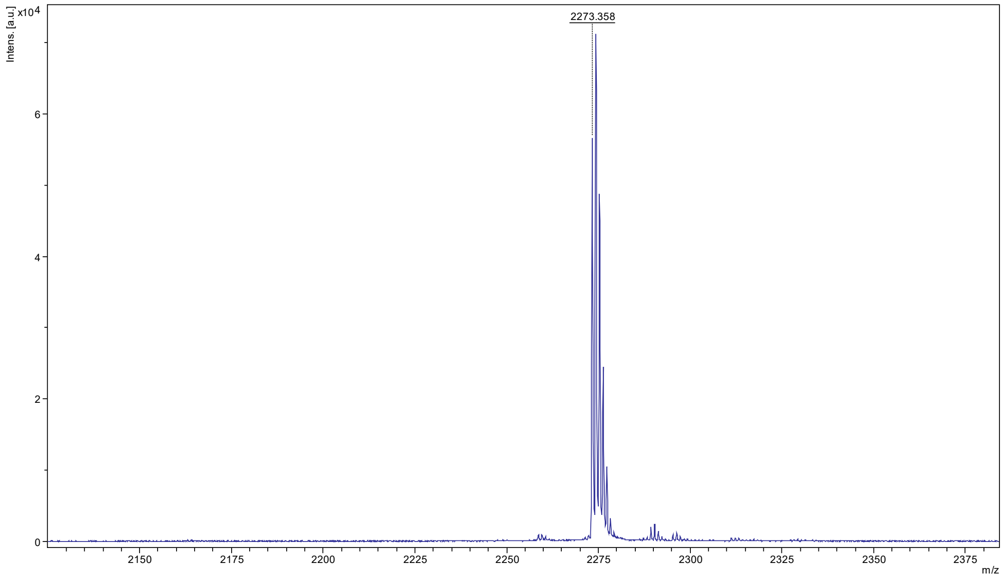

L2

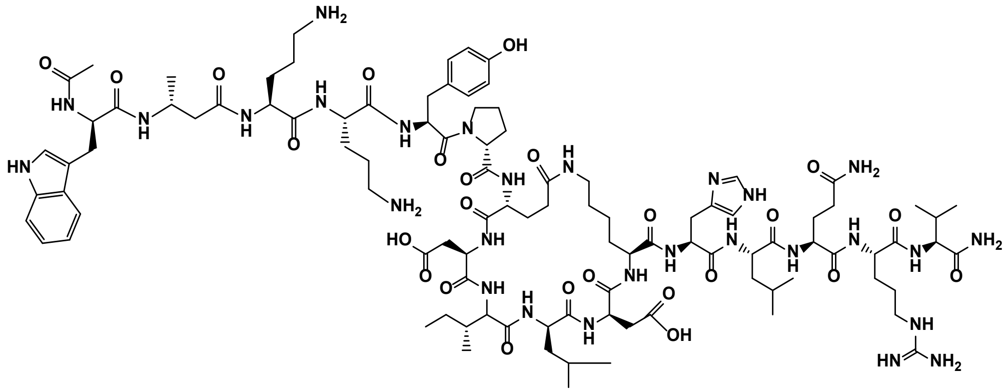

Exact Mass: 2147.1640

Purity assessment by UPLC (214 nm):

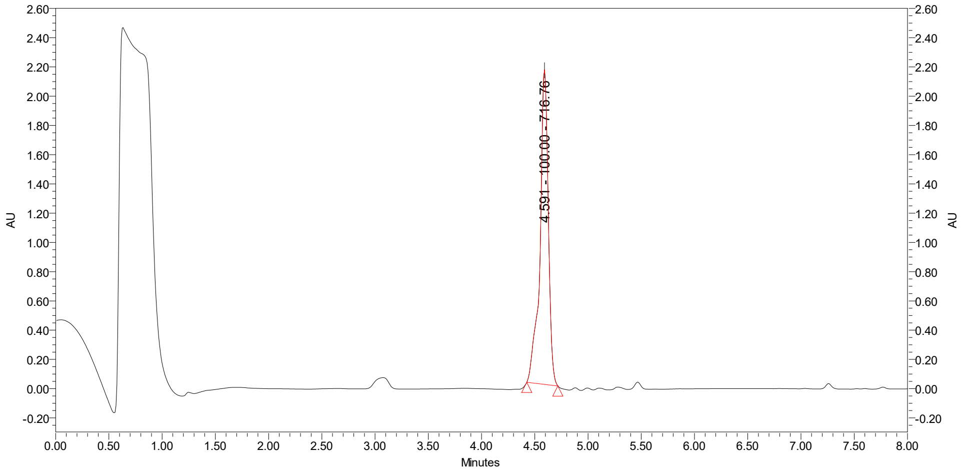

HR-MS:

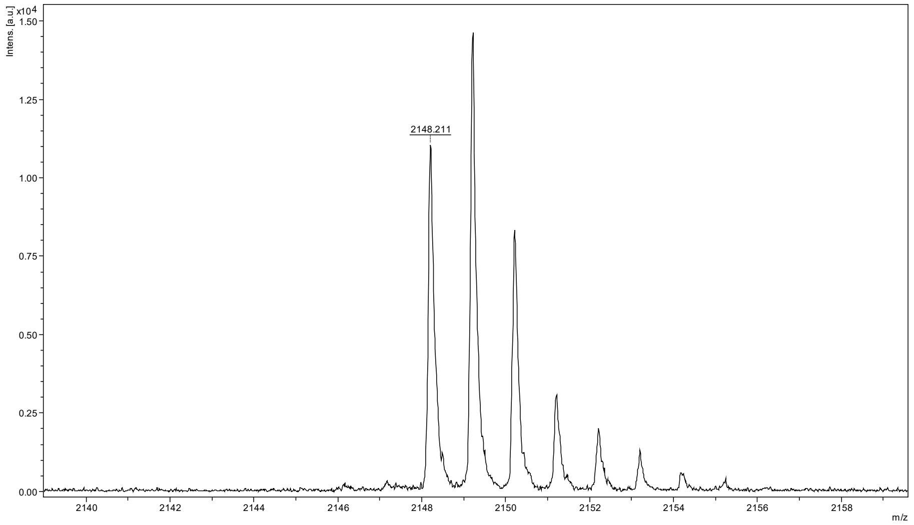

L12

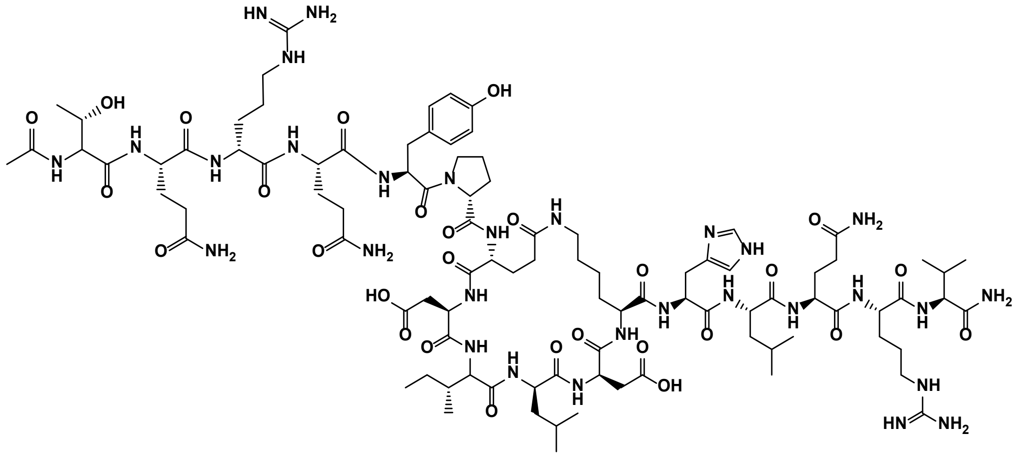

Purity assessment by analytical UPLC (214 nm):

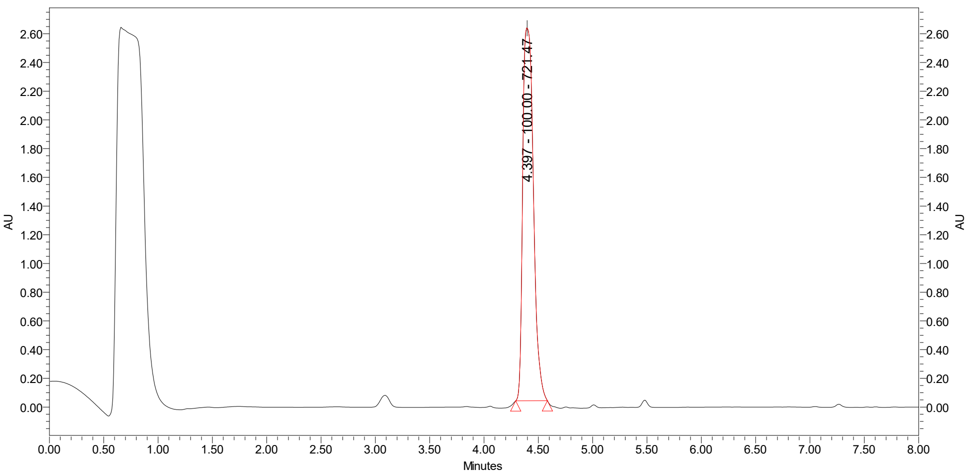

HR-MS:

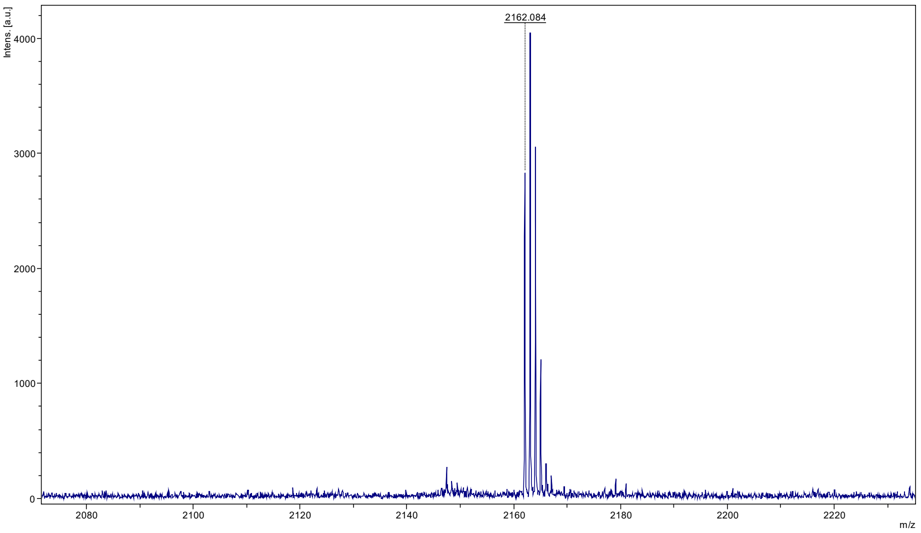

L17

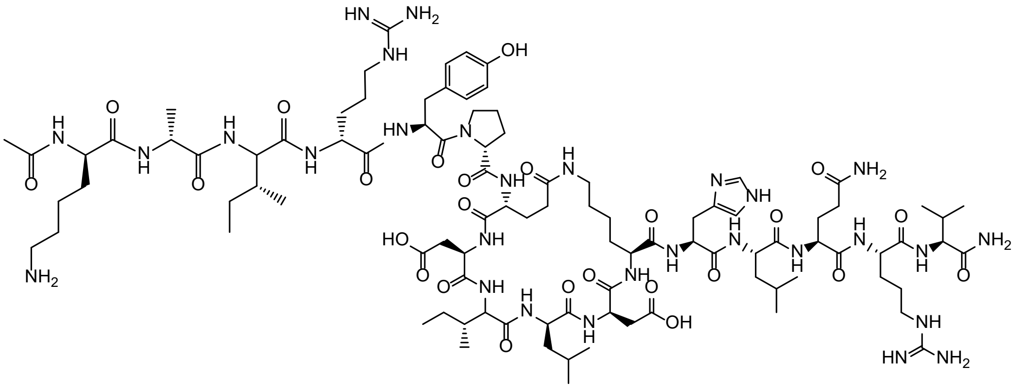

Exact Mass: 2116.1905

Purity assessment by UPLC (214 nm):

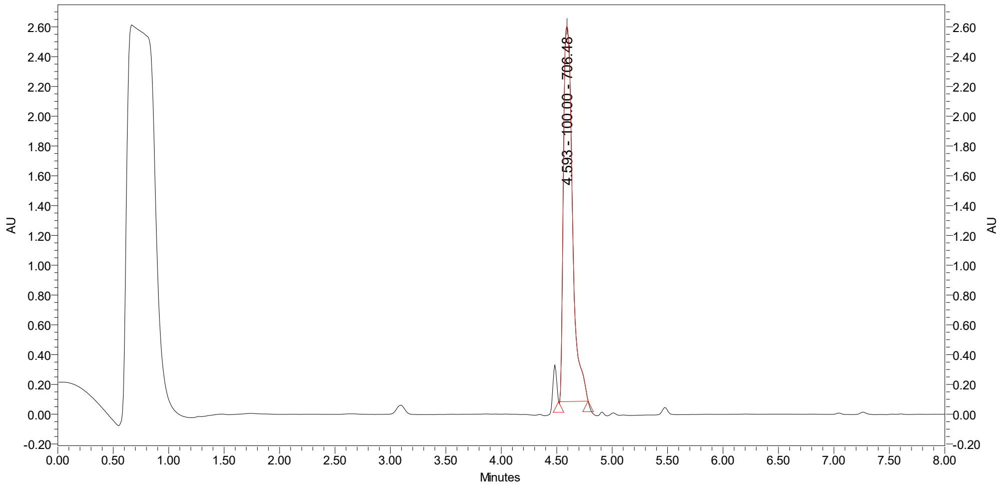

HR-MS:

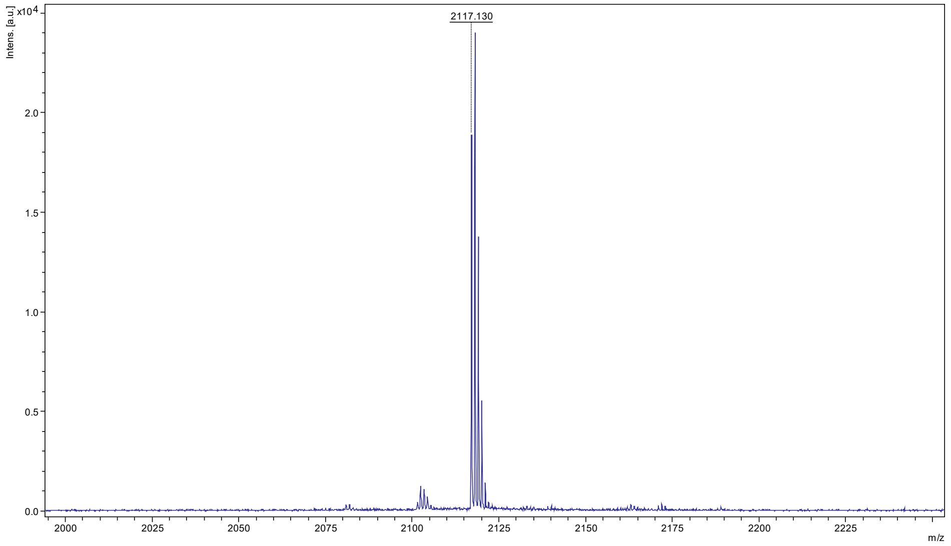

L19

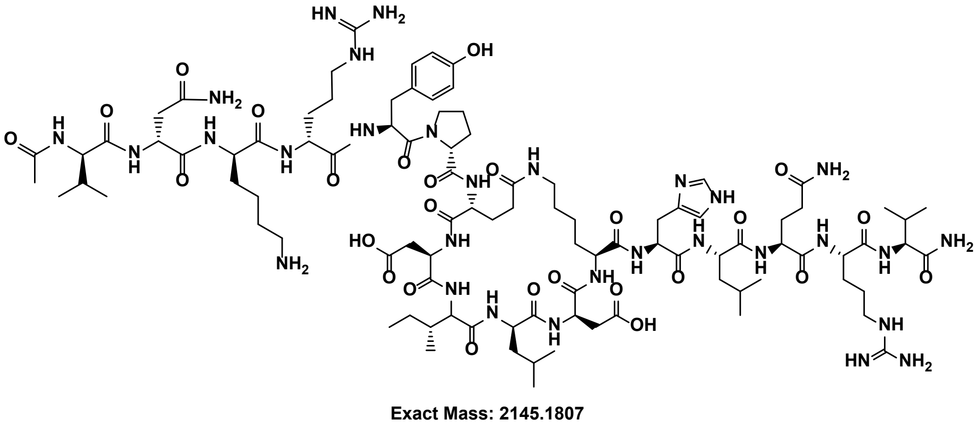

Purity assessment by UPLC (214 nm):

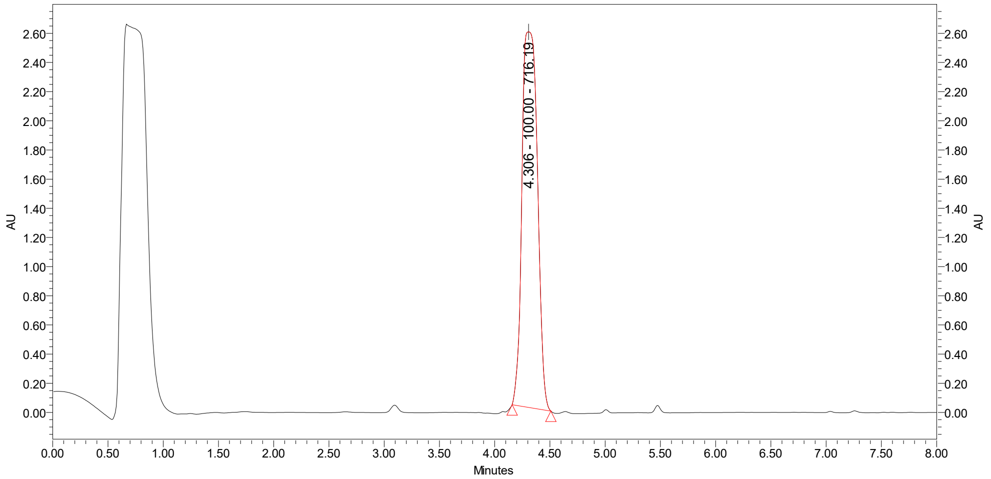

HR-MS:

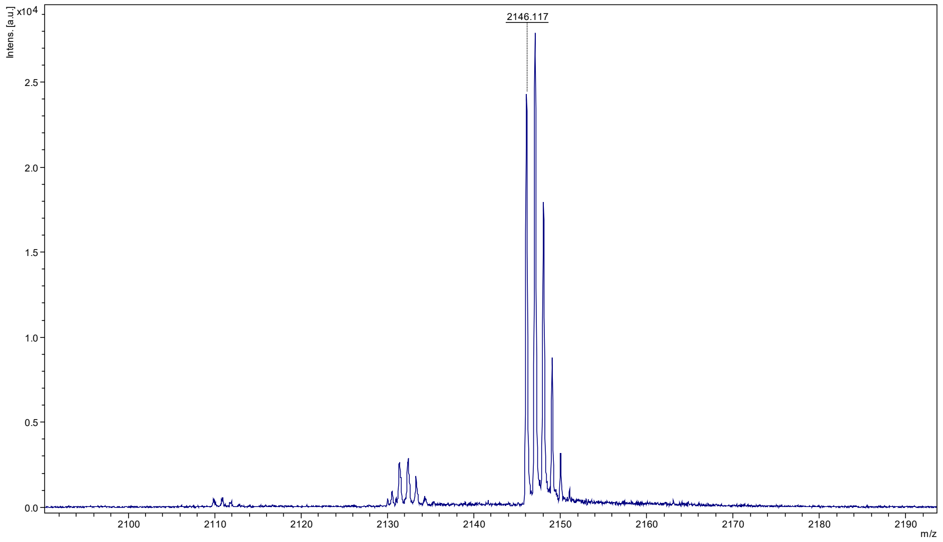

L20

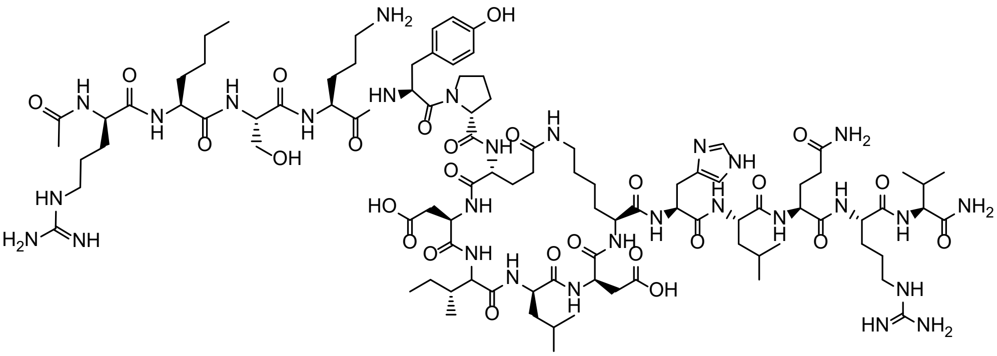

Purity assessment by UPLC (214 nm):

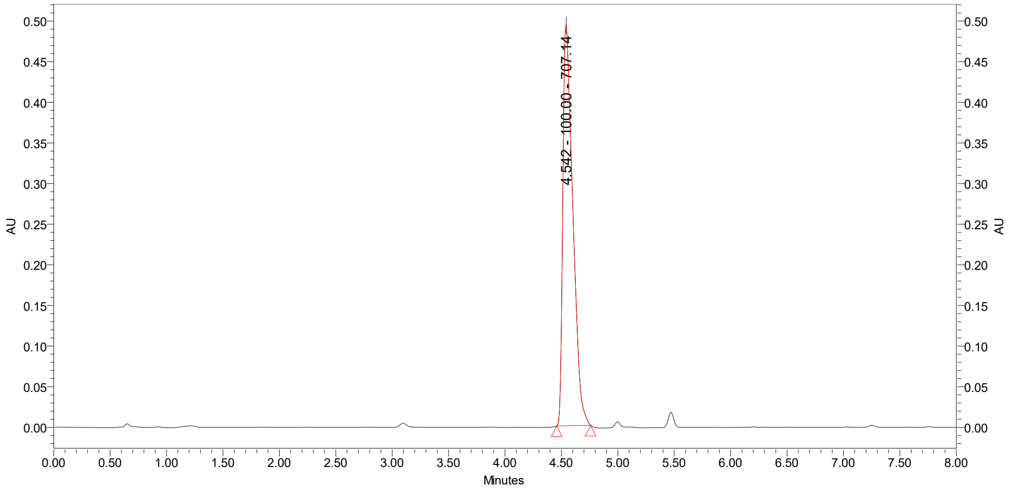

HR-MS:

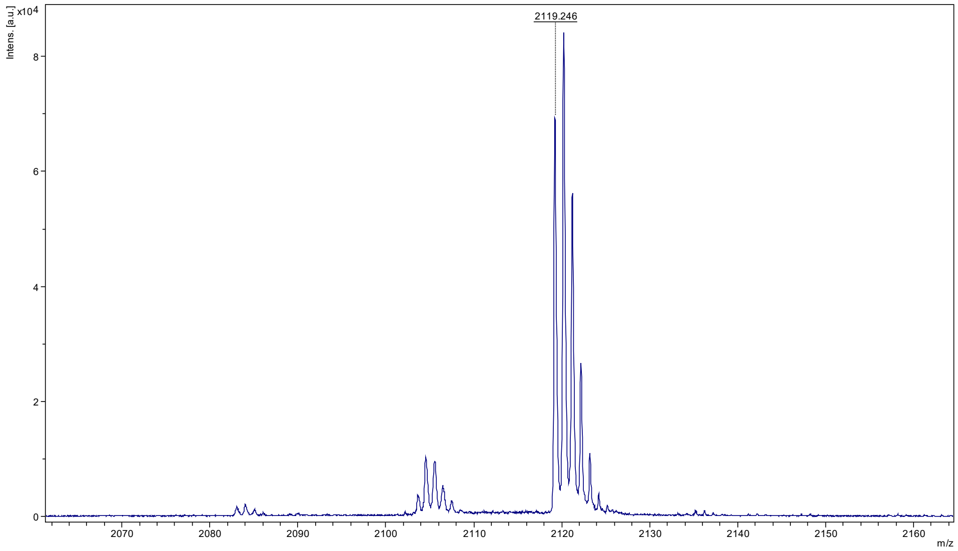

CAL-1

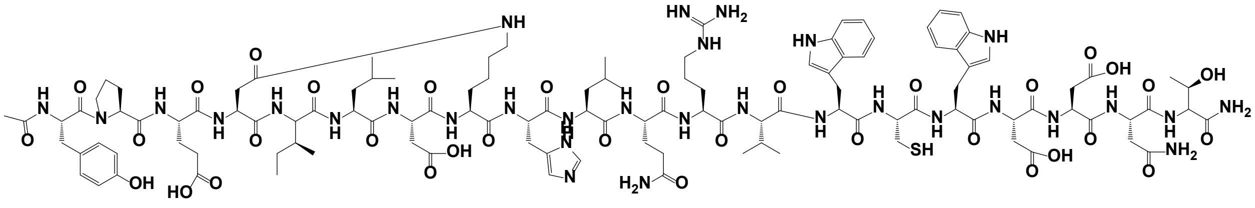

Exact Mass: 2568.1856

Purity assessment by UPLC (214 nm):

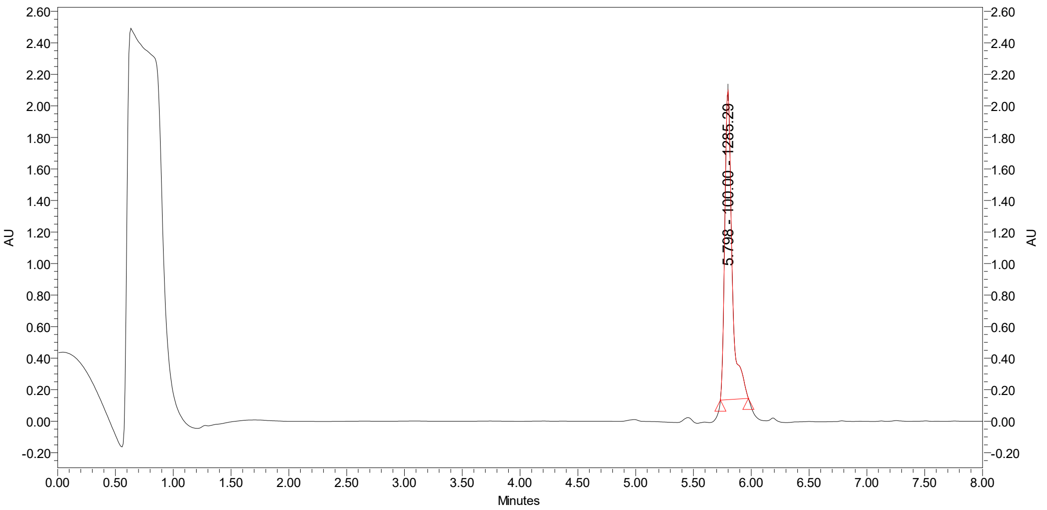

HR-MS:

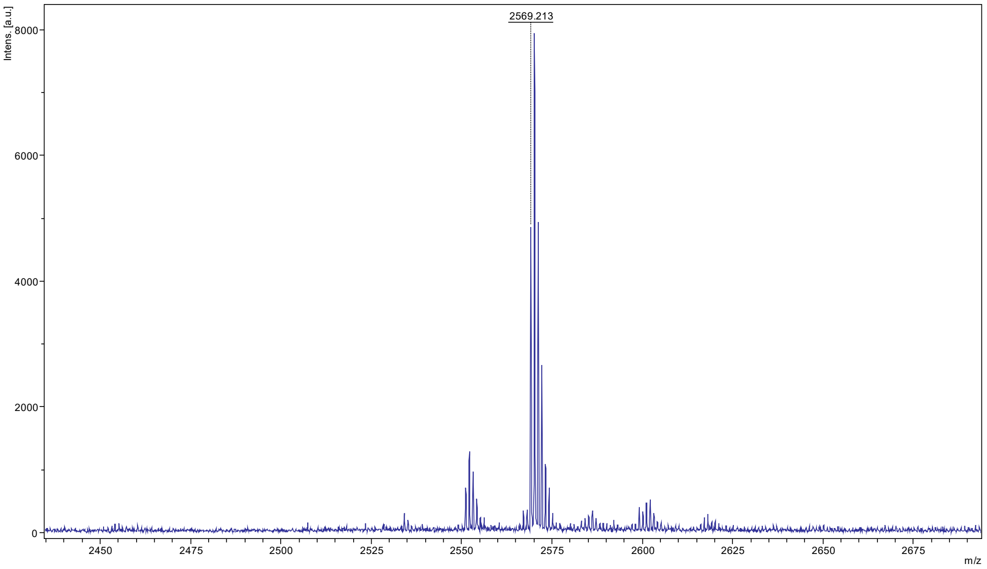

CAL-2

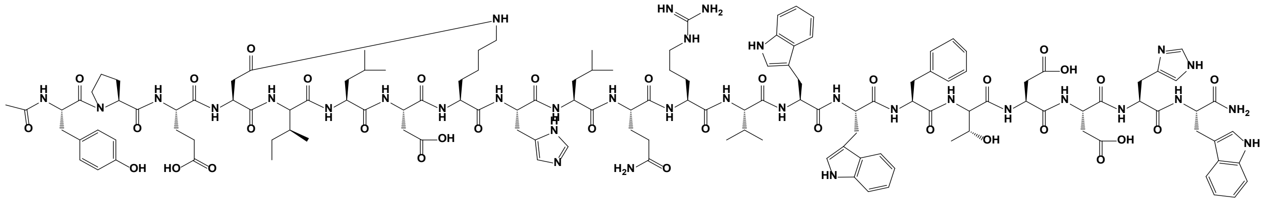

Purity assessment by UPLC (214 nm):

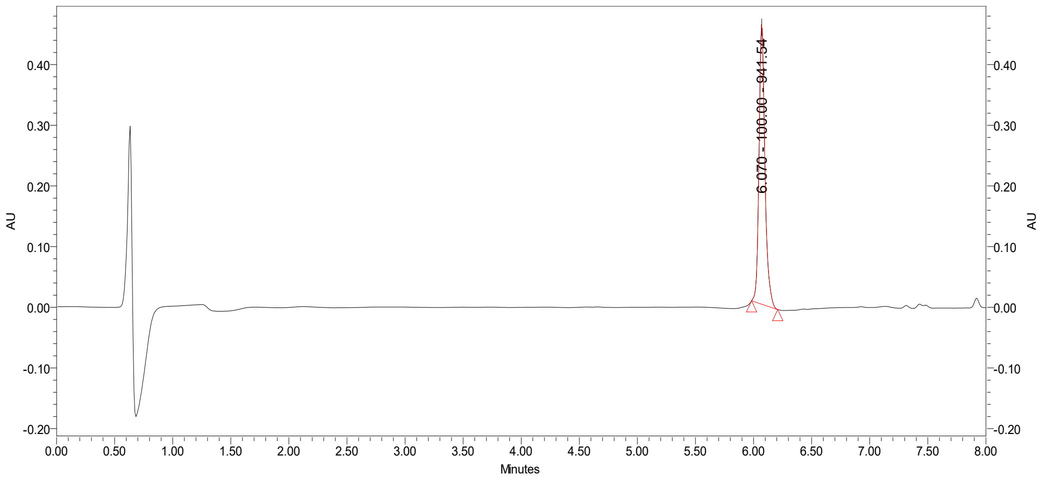

HR-MS:

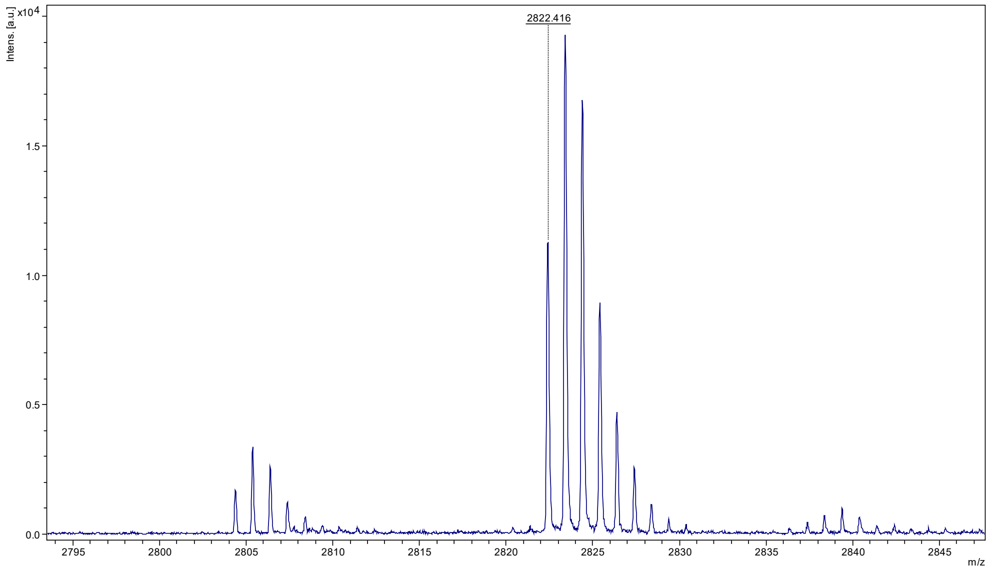

CAL-3

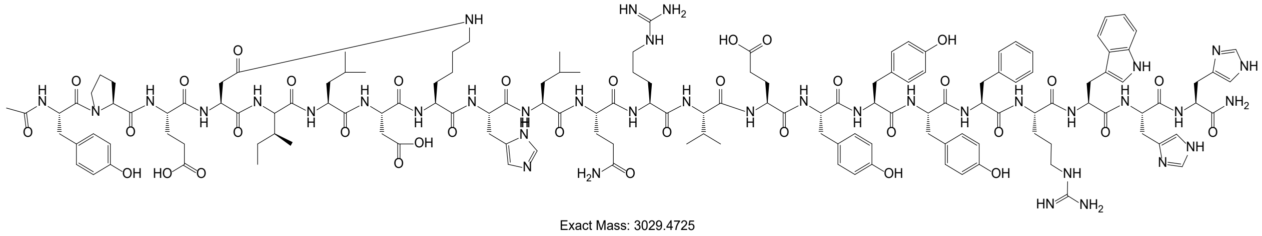

Purity assessment by UPLC (214 nm):

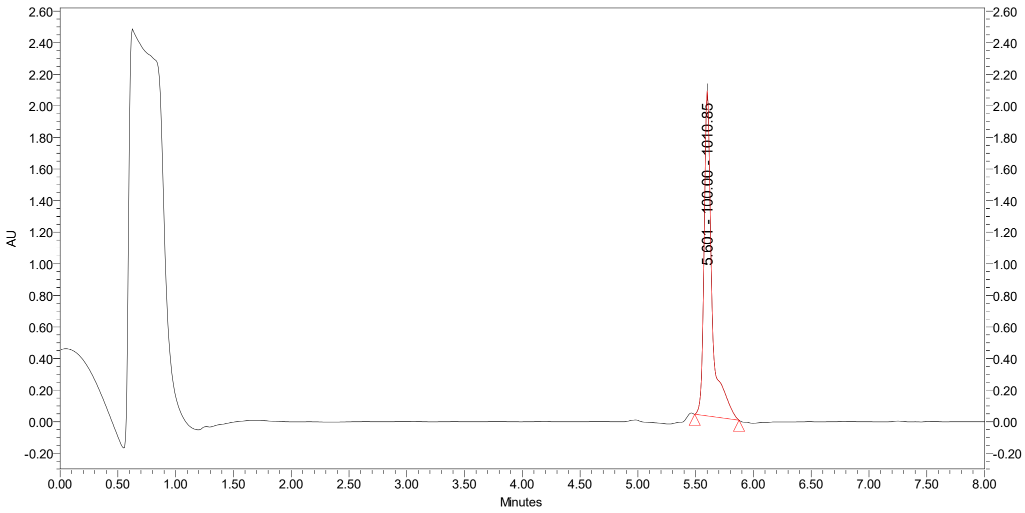

HR-MS:

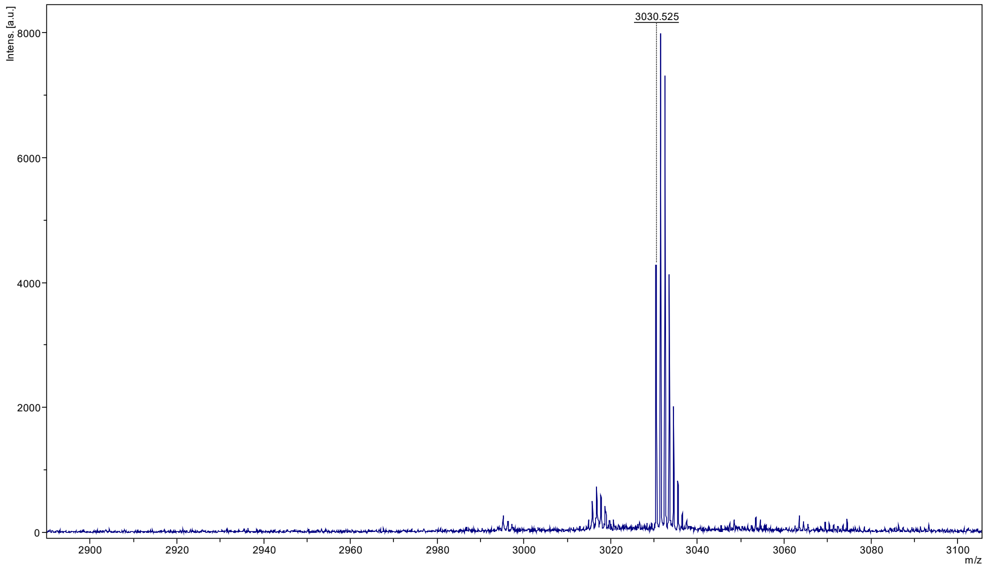

CAL-4

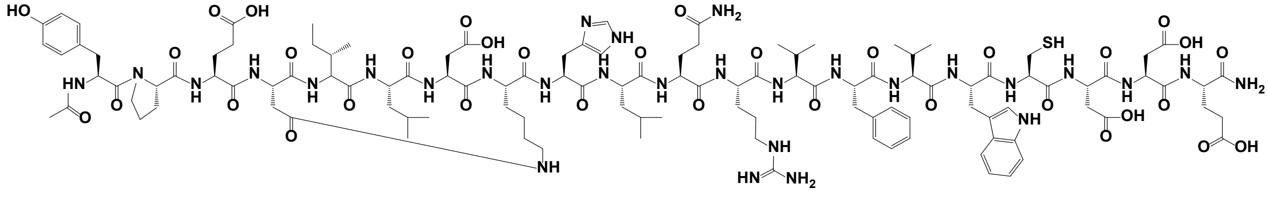

Purity assessment by UPLC (214 nm):

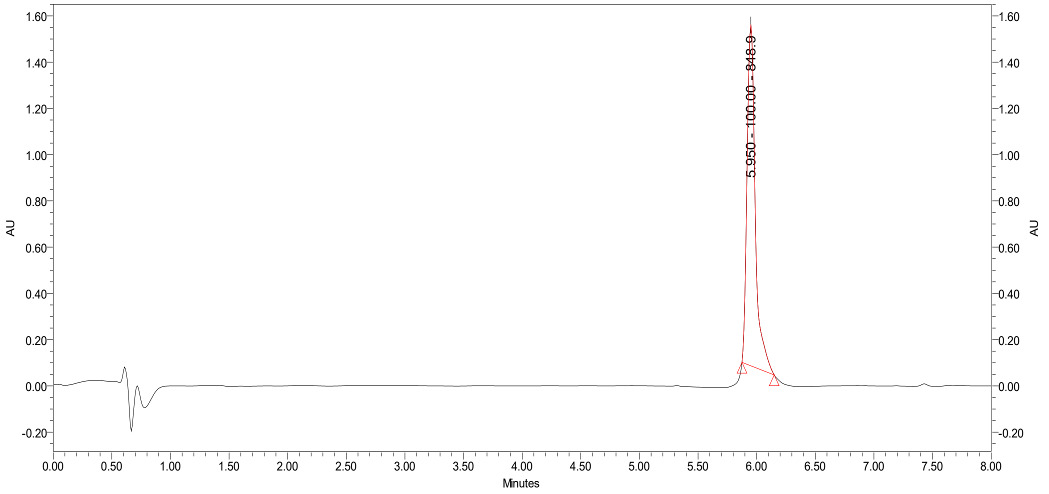

HR-MS:

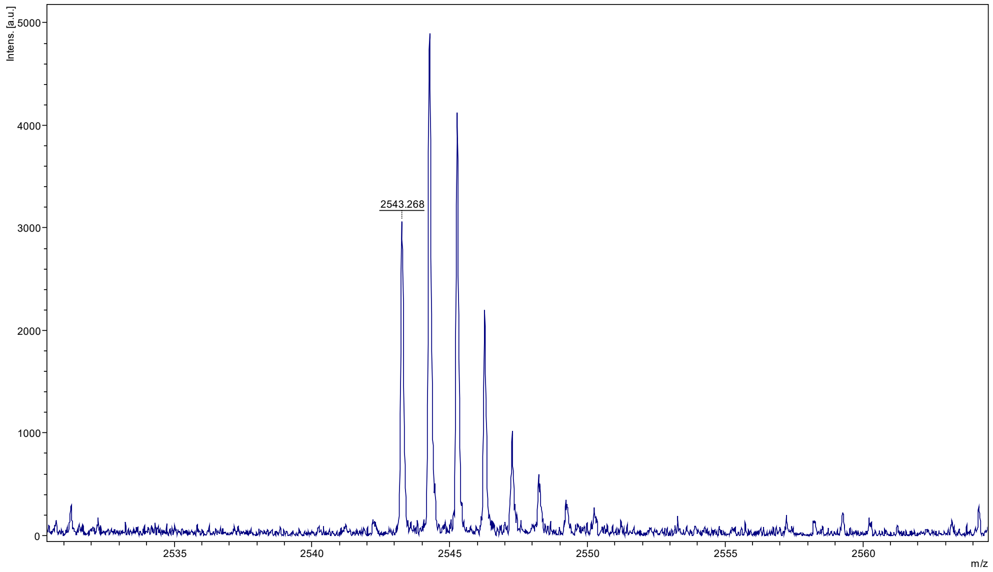

CAL-6

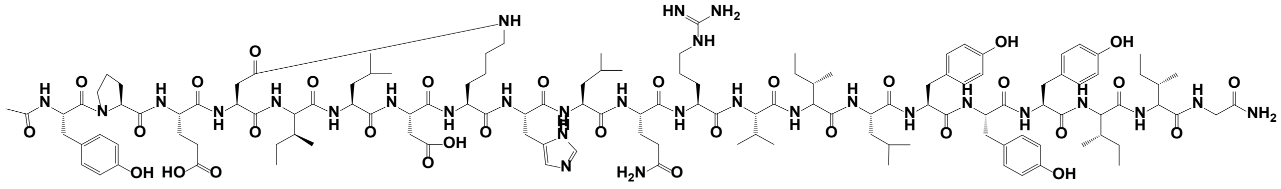

Exact Mass: 2646.4210

Purity assessment by UPLC (214 nm):

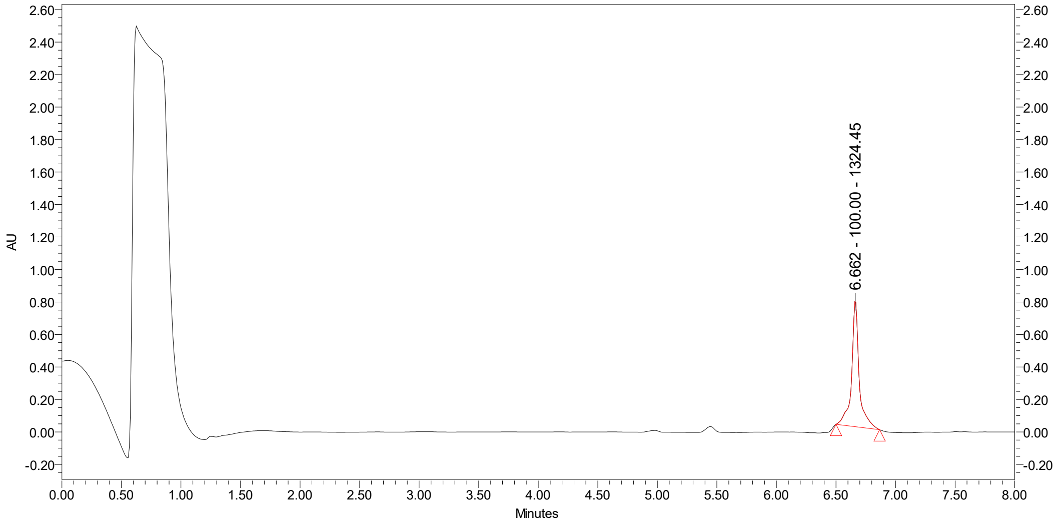

HR-MS:

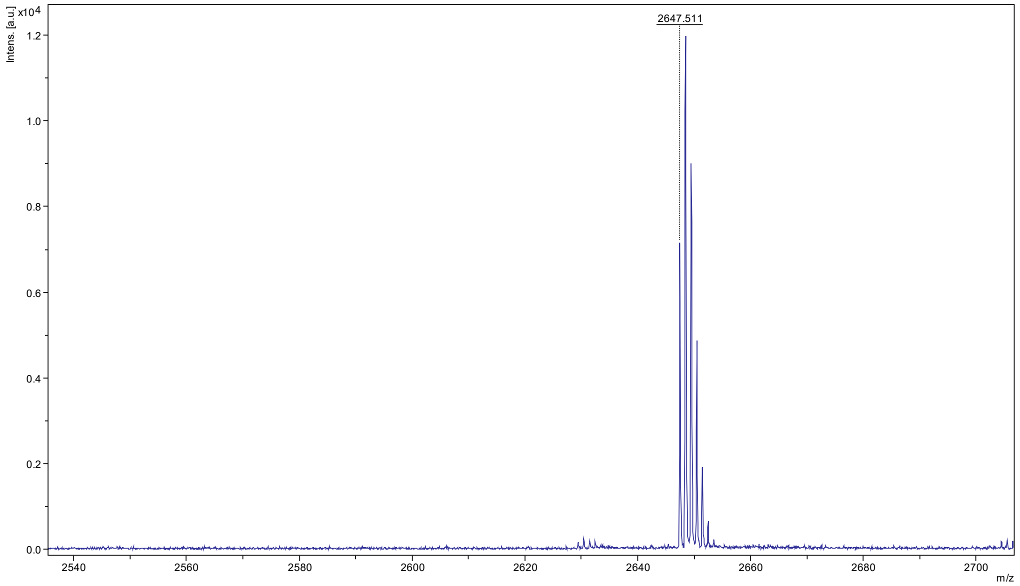

CAL-7

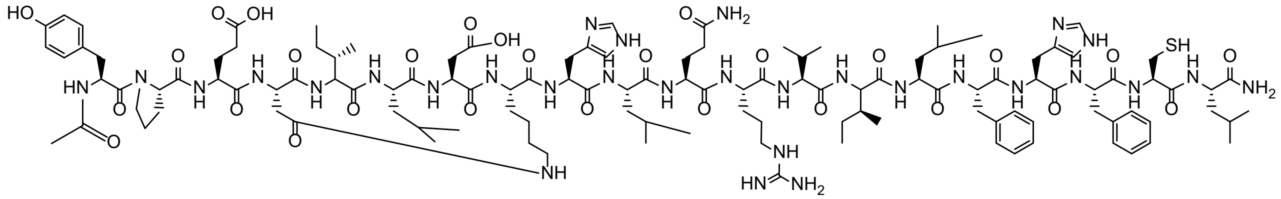

Exact Mass: 2521.3304

Purity assessment by UPLC (214 nm):

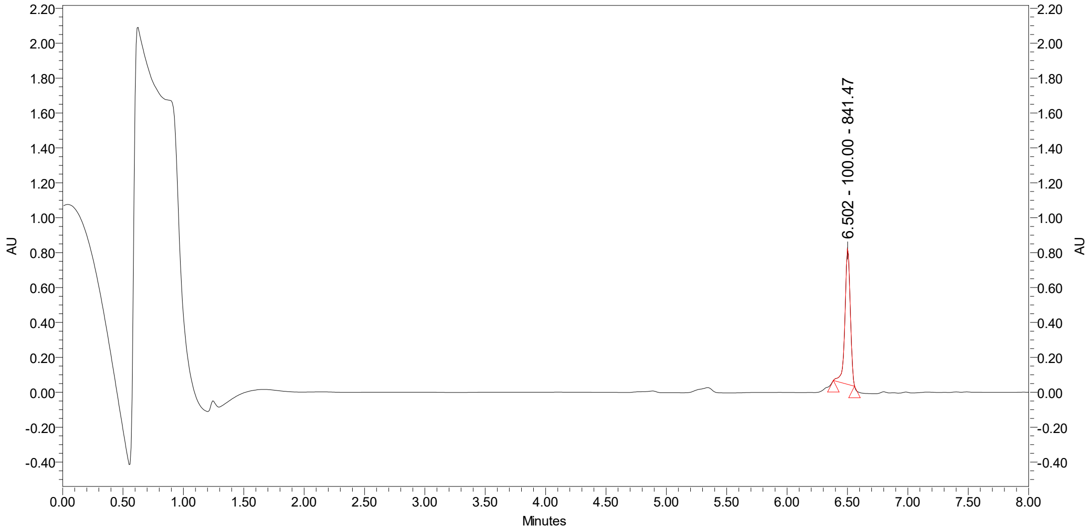

HR-MS:

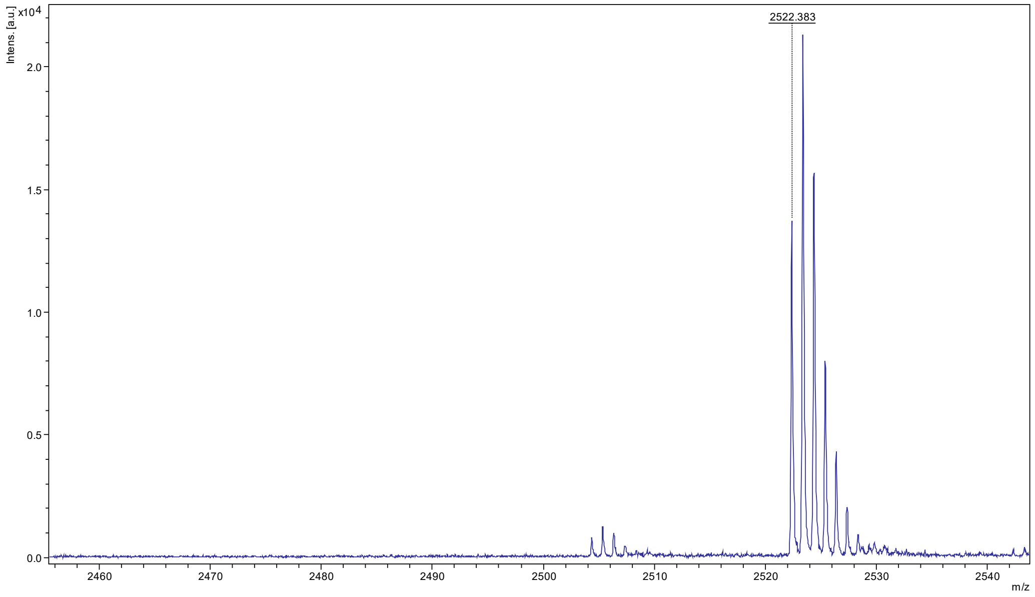

CAL-9

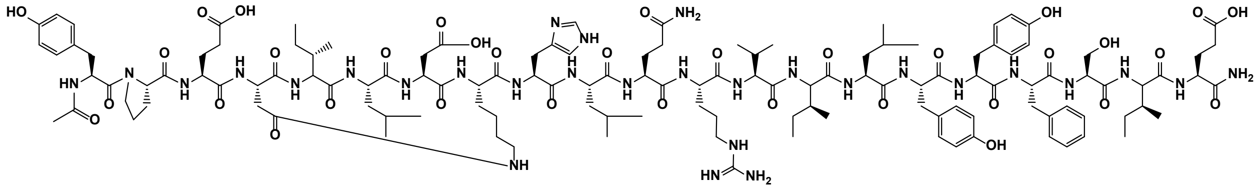

Exact Mass: 2676.3952

Purity assessment by UPLC (214 nm):

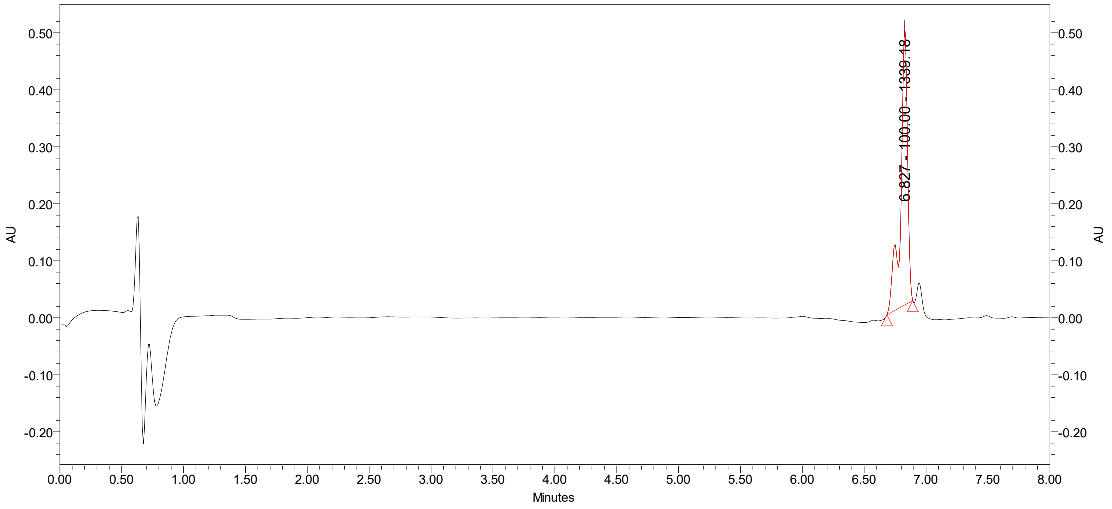

HR-MS:

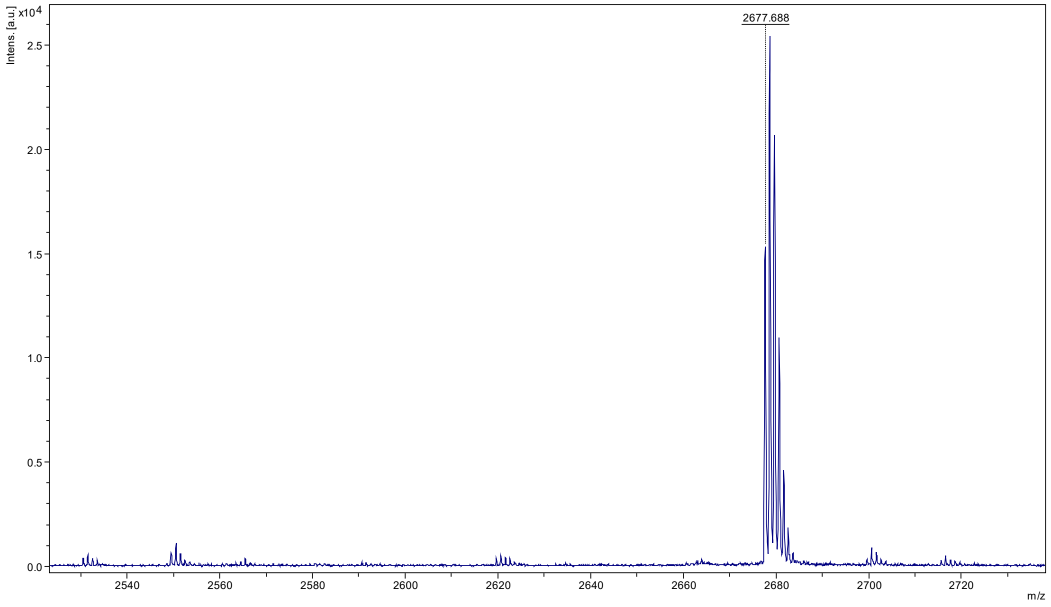

CAL-10

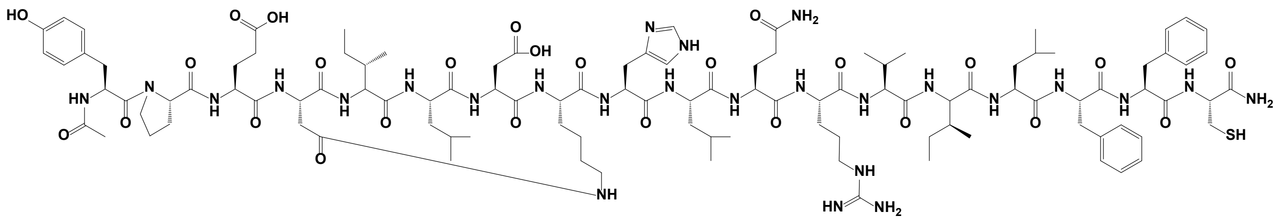

Purity assessment by UPLC (214 nm):

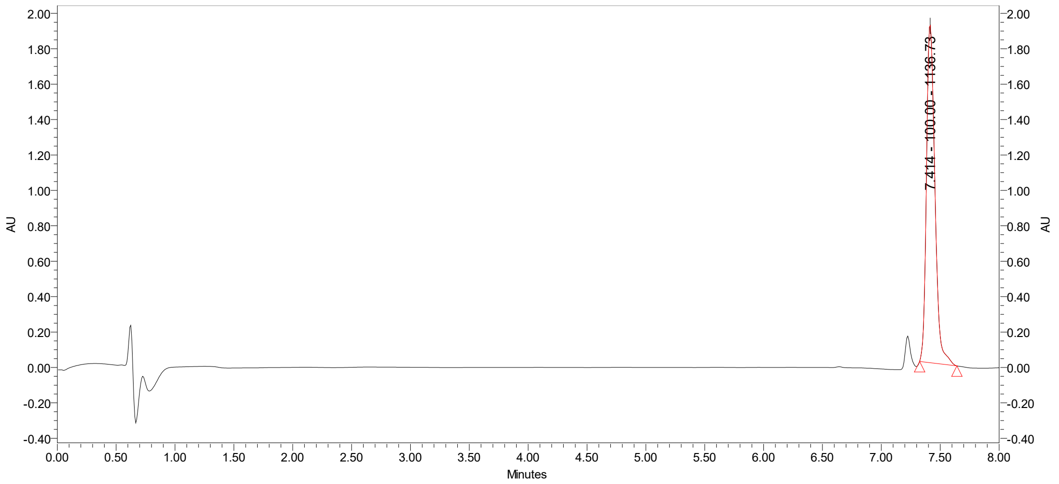

HR-MS:

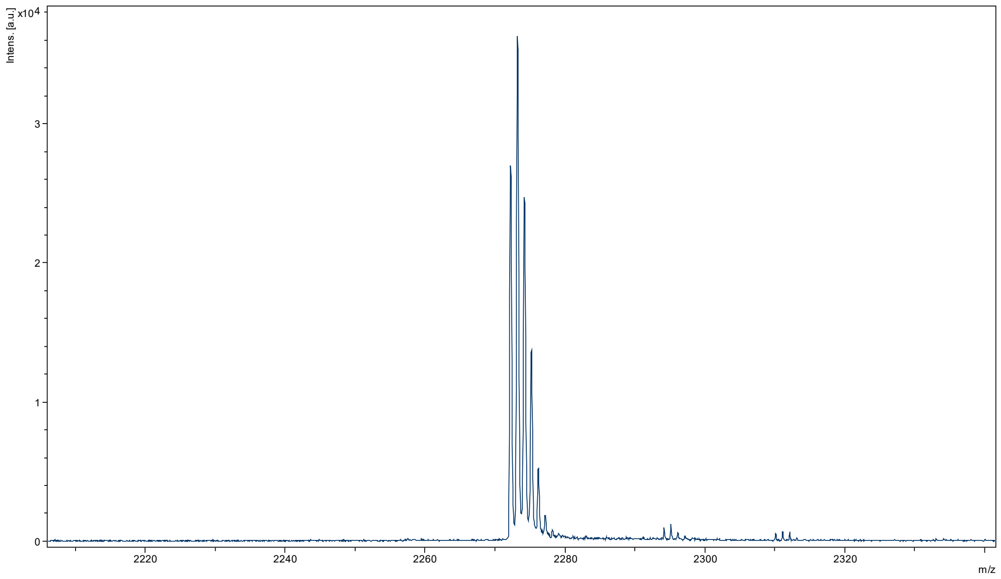

Supplementary Figure 10: UPLC chromatograms and mass spectrometric data of β-catenin binding peptides used in this work. Reversed-phase UPLC was equipped with an analytical C18 column, which was eluted with linear gradients of CH_3CN in water containing 0.05% TFA (monitored at 214 nm). MS was performed on a Bruker ultrafleXtreme MALDI-TOF instrument.

NBD; TALDWSWLQTE:

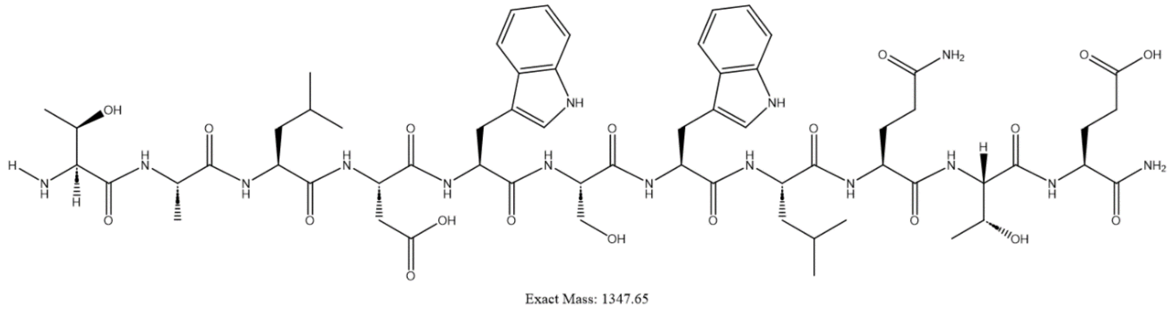

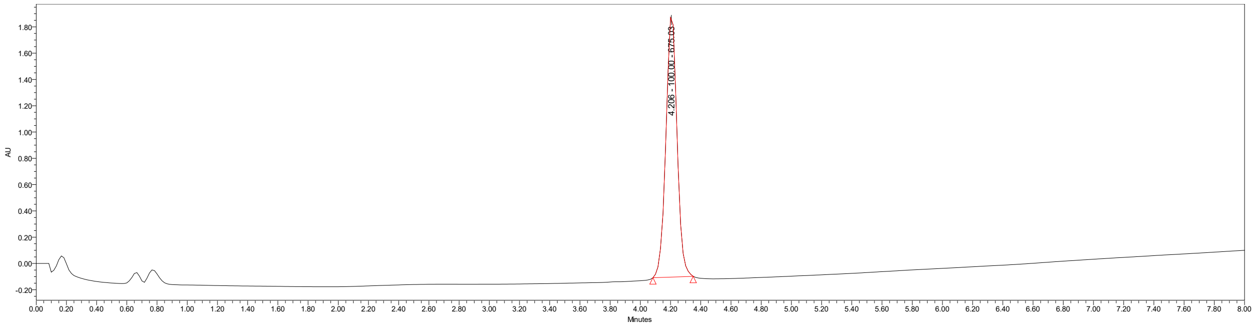

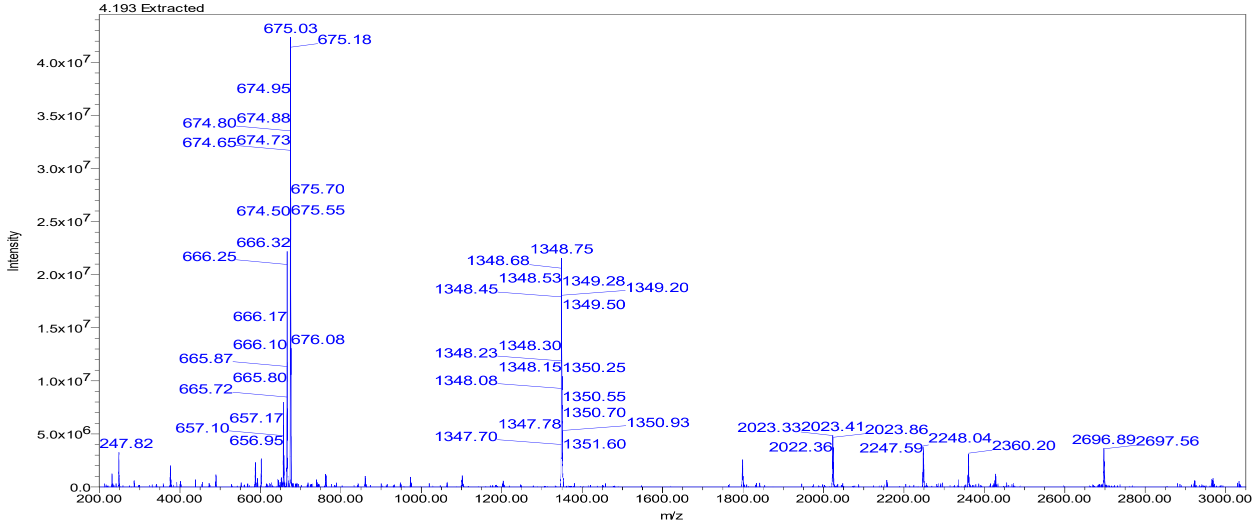

NBD+ ext1; IKKSSTALDWSWLQTE:

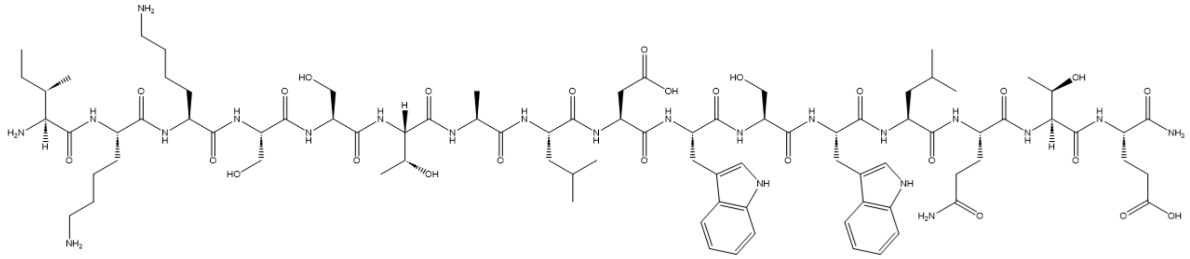

Exact Mass: 1890.98

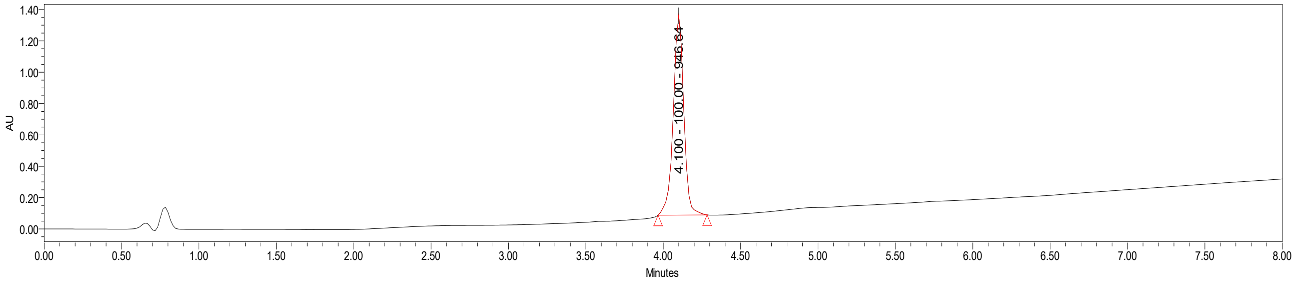

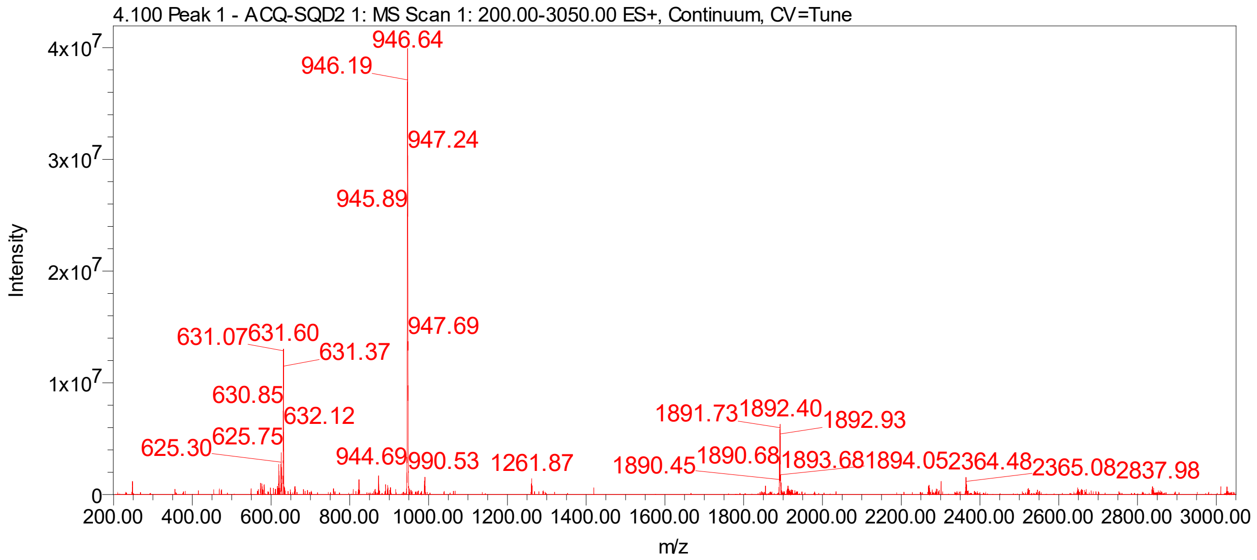

NBD+ ext2; WSHSSHTALDWSWLQTE:

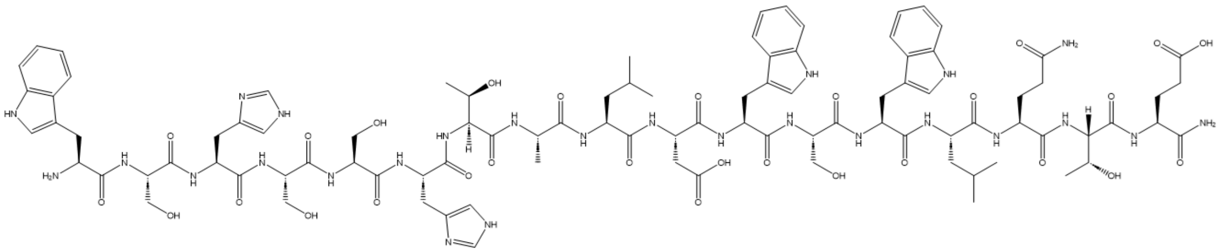

Exact Mass: 2068.94

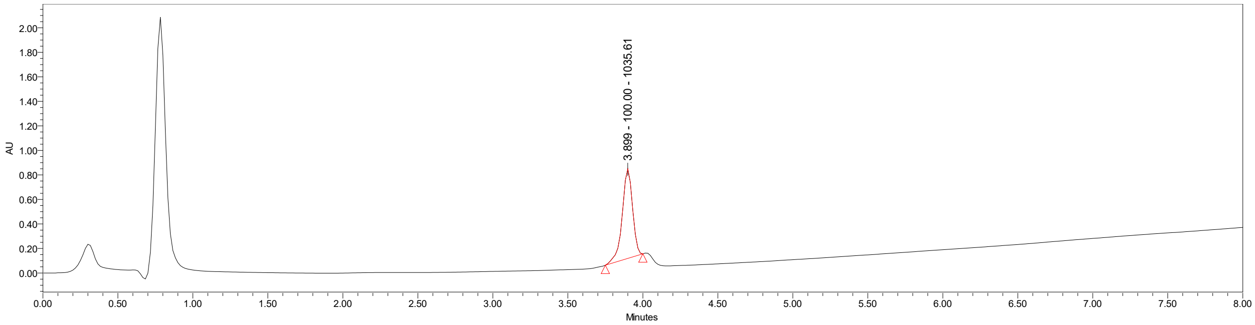

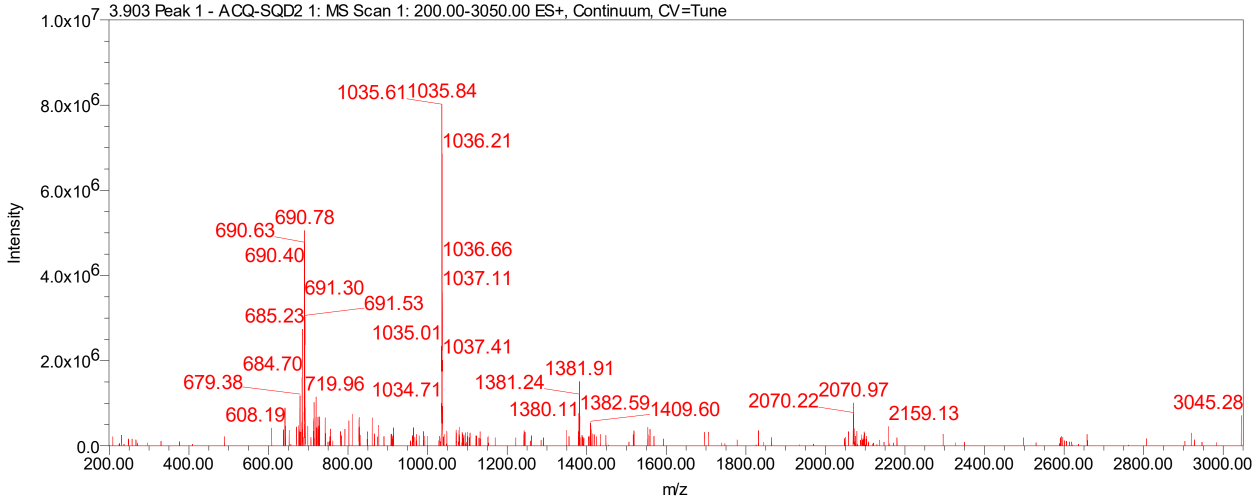

NBD+ ext4; LNQQQSSTALDWSWLQTE:

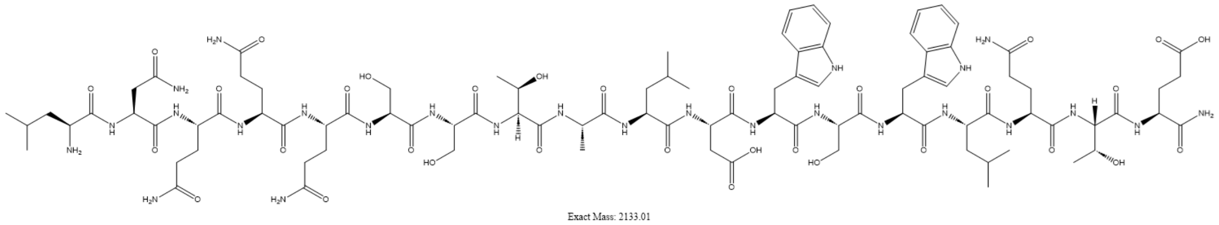

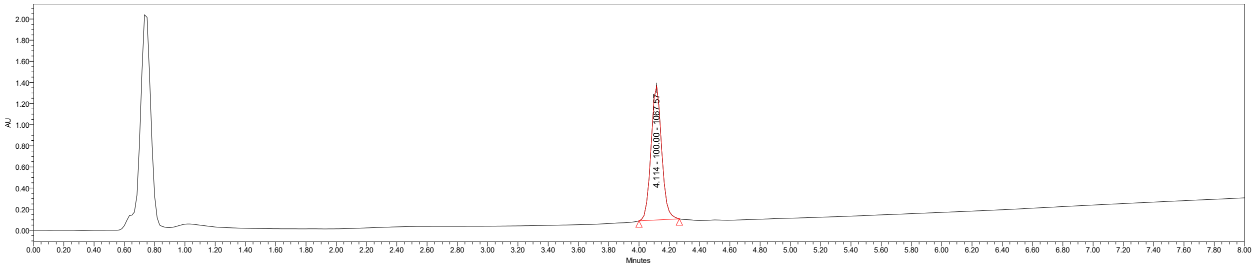

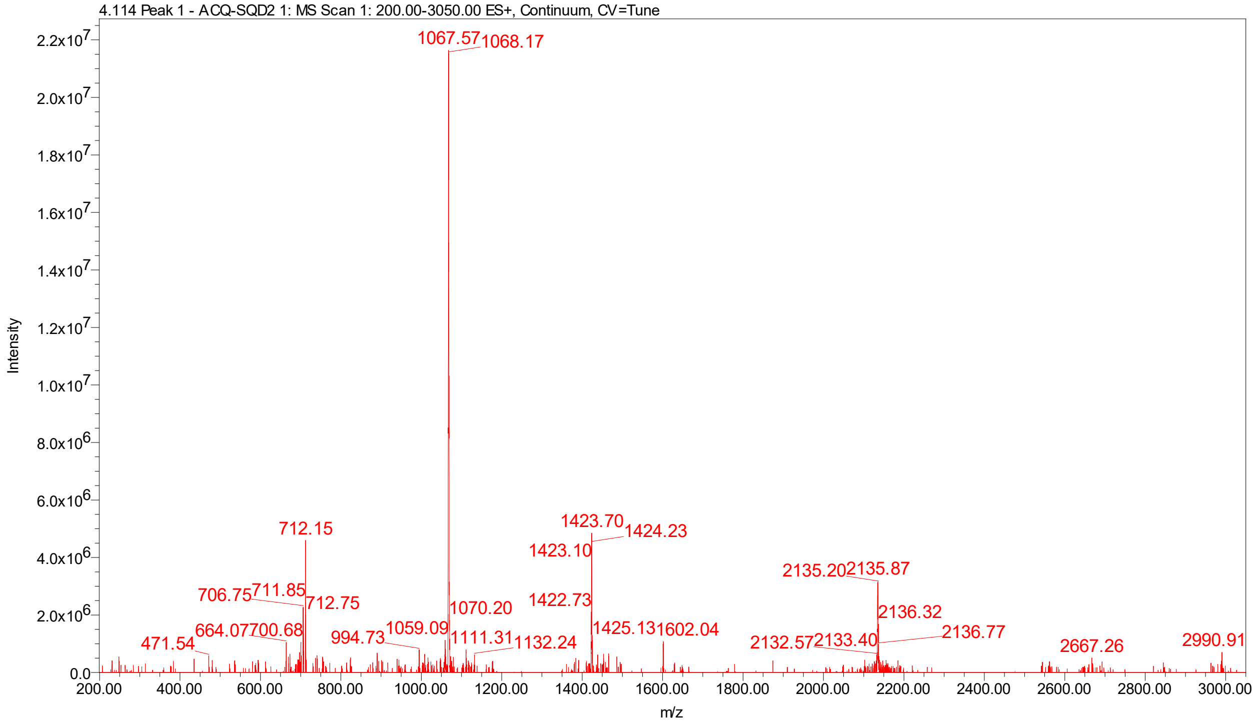

NBD+ ext12; LKSDDSSTALDWSWLQTE:

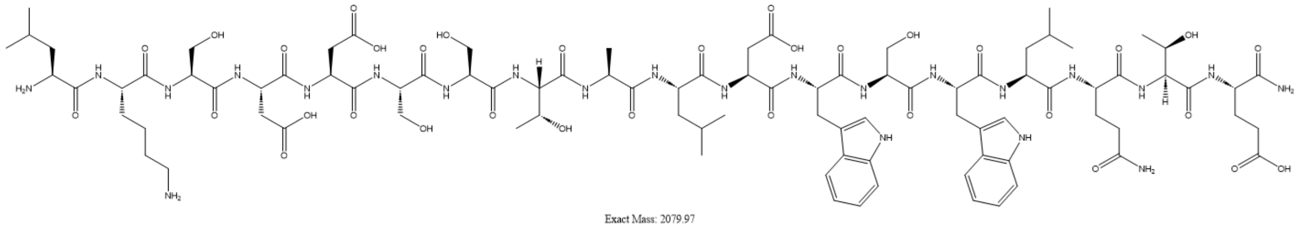

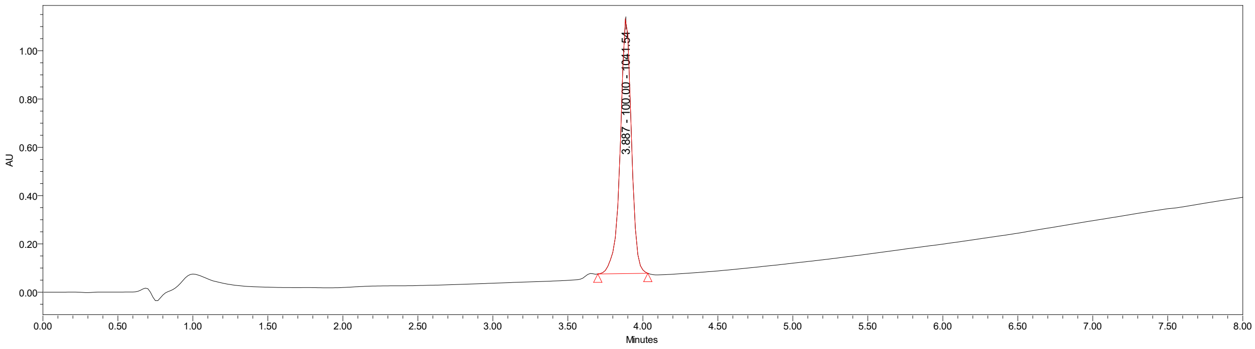

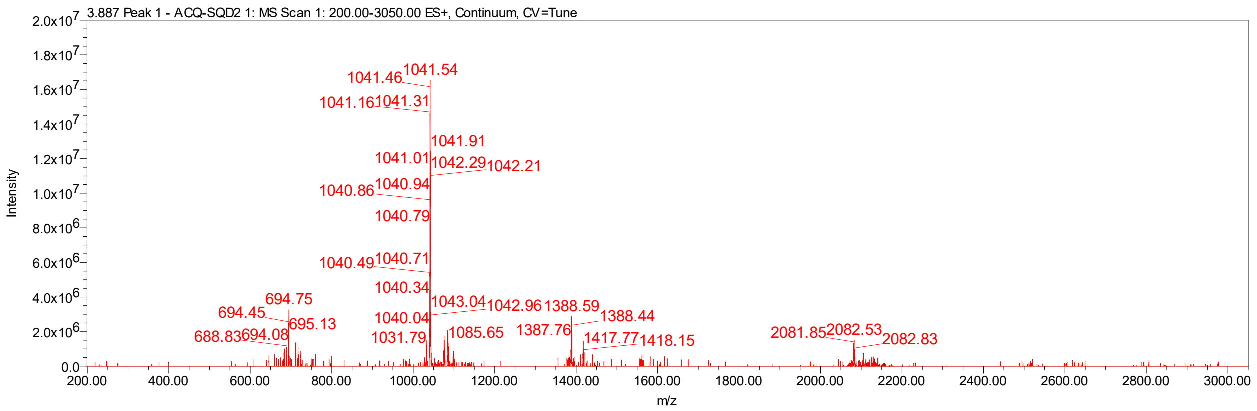

Biotin-IKKβ$_{KK/RR}$; Biotin-β-Ala-PARRSEELVAEAHNLCTLLENAIQDTVREQDSFTALDWSWLQTE

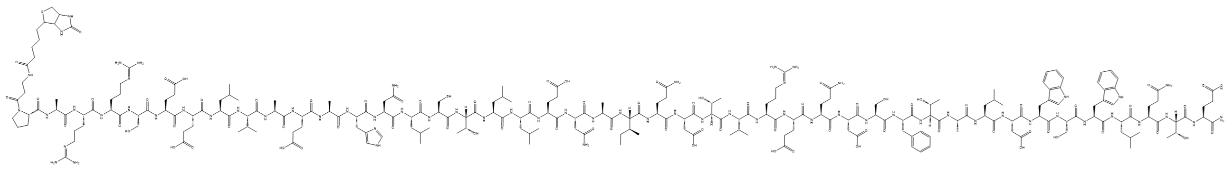

Exact Mass: 5380.59

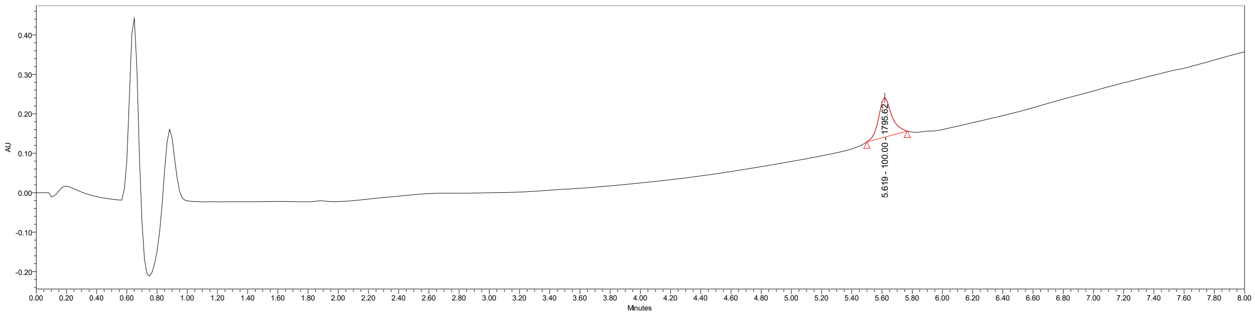

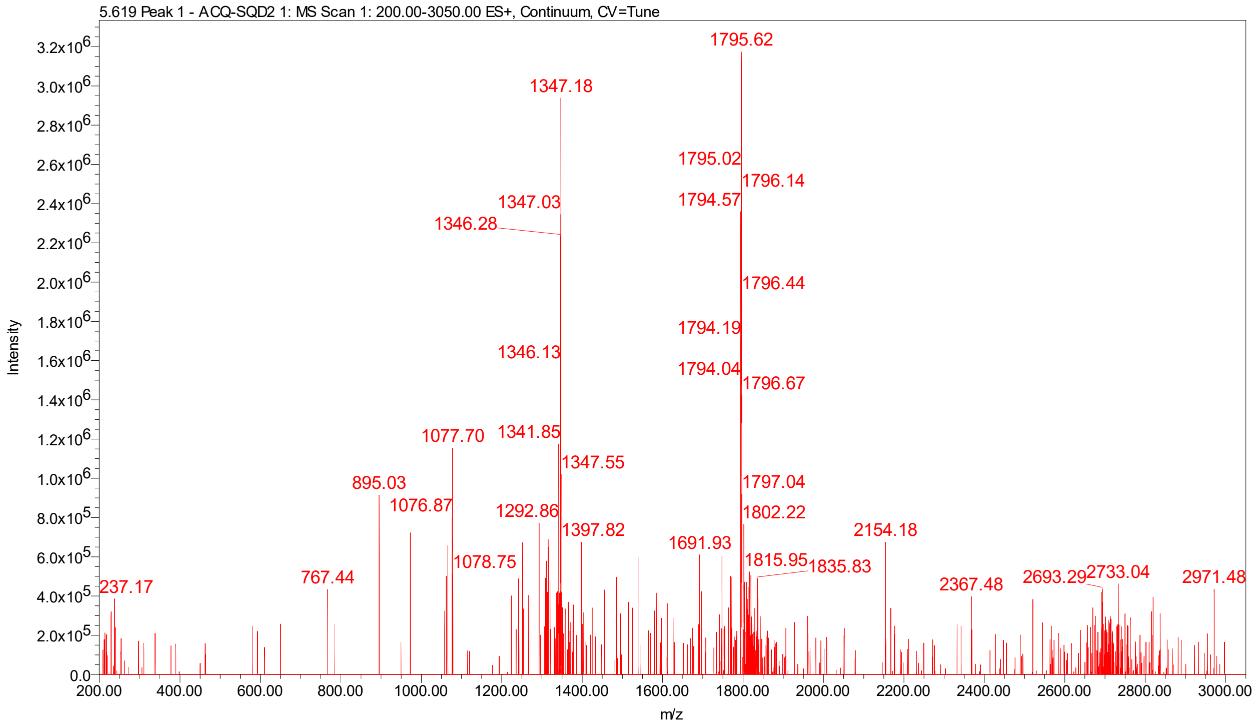

Supplementary Figure 11: UPLC chromatograms and mass spectrometric characterization of NEMO binding peptides used in this work.
